# Managing Uncertainty and Information Dynamics with Graphics-Enhanced TOGAF Architecture in Higher Education

**DOI:** 10.3390/e28030361

**Published:** 2026-03-22

**Authors:** A’aeshah Alhakamy

**Affiliations:** Department of Computer Science, Faculty of Computers and Information Technology, University of Tabuk, Tabuk 47512, Saudi Arabia; aalhakami@ut.edu.sa

**Keywords:** entropy-basedlearner modeling, uncertainty quantification, information dynamics, expected information gain, personalized education, TOGAF enterprise architecture

## Abstract

Adaptive learning at scale requires explicit handling of uncertainty and information flow across diverse educational technologies. This paper proposes a TOGAF-conformant enterprise architecture for the University of Tabuk (UT) that embeds entropy- and uncertainty-aware requirements from the outset and aligns them with institutional goals in teaching, research, and administration. Using the Architecture Development Method (ADM), we map information-theoretic requirements to architectural artifacts across the architecture vision, business, information systems, and technology domains; formally specify core entropy-informed observables, including predictive entropy, expected information gain, workflow variability entropy, and uncertainty hot-spot severity; and define semantic and metadata standards for their near-real-time computation. These indicators are positioned explicitly across the TOGAF domains: business architecture identifies where uncertainty matters, information systems architecture defines the computable data and application representations, technology architecture operationalizes secure and scalable computation, and later ADM phases use the resulting metrics for prioritization and governance. The architecture also establishes governance that ranks initiatives by their expected uncertainty reduction through Architecture Review Board (ARB) decision gates. We address three research questions: (R.Q.1) how to design a TOGAF-conformant architecture for UT that natively encodes uncertainty-aware requirements and aligns with institutional needs; (R.Q.2) how to integrate dispersed data, achieve semantic harmonization, and deliver analytics-ready streams that support information-theoretic indicators for personalization without delay; and (R.Q.3) how to embed IT demand planning in opportunities and solutions and migration planning using uncertainty reduction and expected information gain as prioritization criteria. The resulting architecture offers a university-wide foundation for adaptive learning: it unifies learner and system interaction data under governed schemas, supports low-latency analytics, and formalizes decision processes that treat uncertainty as a primary metric. Though learner-level operational validation is future work, the design establishes the technical and organizational foundations for responsible, large-scale deployment of entropy-driven learner modeling, content sequencing, and feedback optimization.

## 1. Introduction

The rapid growth of digital technologies has fundamentally reshaped how learning is delivered, experienced, and governed, amplifying the scale, speed, and diversity of educational data [1]. In these data-intensive settings, uncertainty permeates decision-making at multiple levels, from learner modeling and adaptive content delivery to institutional planning and quality assurance [2]. Entropy, as a theoretically grounded measure of uncertainty and information flow, provides a unifying framework for characterizing variability in learner states, diagnosing process instability, and guiding data-driven personalization [3]. For this framework to be rigorous at institutional scale, entropy and related information-theoretic quantities must go beyond conceptual metaphors: they must be formally specified, computationally embedded in enterprise data flows, and explicitly situated within the architectural domains that govern their use. Implementing entropy-informed adaptive learning at institutional scale thus requires more than isolated analytics; it calls for an enterprise architecture (EA) that systematically aligns educational objectives with information assets, applications, and technologies, while treating uncertainty and information dynamics as primary design concerns [4].

Higher education institutions (HEIs) experience this imperative with particular intensity. Escalating competitive pressures and increasingly stringent stakeholder expectations require universities to provide coherent, interoperable services that enable personalized learning and continuous quality enhancement [5]. In the absence of a strategically designed architectural backbone, fragmentation across processes, data semantics, and governance structures can obscure information flows, constrain the observability of learner trajectories, and impede the implementation of uncertainty-aware interventions. Enterprise architecture (EA) addresses these challenges by offering a systematic approach to aligning institutional strategy with information technology capabilities, reducing operational complexity, and coordinating a shared blueprint for adaptive learning ecosystems [2,4]. In this context, the central challenge is not only to acknowledge uncertainty, but also to define where it arises, how it is computed from institutional evidence, and how it is used in business, system, technology, and governance decisions.

This study investigates the University of Tabuk (UT), Saudi Arabia, as an empirical context for operationalizing an entropy-centric enterprise architecture (EA). UT’s information ecosystem encompasses both core academic activities and auxiliary services and is characterized by heterogeneous information systems at varying levels of maturity.

Building on The Open Group Architecture Framework (TOGAF) [6], we design an architecture that integrates strategic business alignment with explicit modeling of information dynamics. TOGAF’s Architecture Development Method (ADM) [7] is adapted to: (i) elicit pedagogical and operational drivers for personalization; (ii) design data, application, and technology architectures that preserve, expose, and orchestrate information relevant to learner state estimation; and (iii) institutionalize governance mechanisms for uncertainty quantification, including entropy-based indicators of data quality, process variability, and model confidence [8]. More specifically, the proposed adaptation formalizes a set of entropy-informed observables—including predictive entropy, expected information gain, workflow variability entropy, and uncertainty hot-spot severity—and embeds them across the TOGAF lifecycle so that they can be defined in the architecture vision, localized in business architecture, computed through information systems architecture, operationalized in technology architecture, and used for prioritization and monitoring in later ADM phases.

The flexibility of TOGAF and its status as an open standard make it well suited to incrementally embedding entropy-aware diagnostics and decision-support into existing university processes while controlling costs and operational risks [4]. This positioning matters because this study does not claim a full deployment of learner-level entropy analytics; rather, it offers a formal, institutionally grounded architectural framework in which these indicators become computable, governable, and decision-relevant.

By organizing enterprise integration around information-theoretic observables—such as entropy of learner knowledge states, assessment-based information gain, and workflow variability—the architecture creates the infrastructure needed for scalable adaptive learning. It frames the university as a cyber-socio-technical system whose data flows across academic, student-support, and administrative domains are intentionally designed to expose uncertainty, guide interventions, and enable systematic personalization. Accordingly, the manuscript contributes an enterprise architecture design and an information-theoretic formalization layer that specify how uncertainty-aware indicators are defined, computed from governed UT data pipelines, and linked to architectural and governance decisions. This work makes three primary contributions:

- Real-world entropy-aware EA. A university-wide architectural blueprint for the UT that applies TOGAF to enhance technological competitiveness while instrumenting the institutional information environment for entropy-based learner modeling and adaptive service delivery. The architecture aligns institutional strategy with governed acquisition of interaction data at temporal and semantic granularities suitable for uncertainty quantification [5].
- Methodological integration. A domain-specific adaptation of TOGAF’s ADM phases for higher education, with concrete architectural artifacts that locate uncertainty hot spots, define entropy-based KPIs, and align business objectives with data, application, and technology architectures for personalization, making uncertainty an explicit, traceable requirement from strategic vision to governance [2,4,8]. The present manuscript strengthens this contribution with formal mathematical definitions, computational derivation rules, and explicit positioning of the indicators across TOGAF domains.
- Sustained evolution. A governance framework and evolutionary roadmap that elevate variability metrics and information-dynamics indicators to primary decision criteria for portfolio management, guiding investment, migration, and risk decisions while maintaining architectural fitness-for-purpose amid changing institutional and technological conditions [4].

## 2. Background and Related Work

TOGAF is a widely used EA methodology for designing, planning, implementing, and governing EIA [9]. Introduced in 1995, it builds on TAFIM and incorporates ideas from IAF [10]. In this study, EIA is treated both as a means of aligning information assets with organizational strategy and as a mechanism for modeling uncertainty, information flow, and variability in learning ecosystems.

EIA comprises the structures, processes, and standards by which organizations govern and use information resources to achieve strategic goals. Its core concerns include information requirements, data standards and semantics, and governance mechanisms that ensure accuracy, consistency, timeliness, and accessibility [11]. In education, these shape the observability of learner states, the reliability of learning analytics, and the responsiveness of personalized learning systems. From an information-theoretic perspective, coherent data architectures enable entropy-based indicators, while application and technology architectures support their capture, processing, and near-real-time use.

EIA spans several interdependent domains. Data architecture defines structures, relationships, and semantics that make datasets comparable and combinable, enabling uncertainty and information-gain calculations across platforms. Application architecture integrates services such as assessment, LMS, and advising so decision-relevant signals are preserved rather than siloed. Technology architecture supplies the compute, storage, network, and analytics needed to process high-velocity educational data in near real time. Information governance and metadata management cut across these domains to enforce integrity, confidentiality, availability, compliance, security, shared vocabularies, and lineage, making uncertainty estimates interpretable and auditable [12]. Together, these capabilities enhance decision quality, operational efficiency, and institutional competitiveness, while enabling more trustworthy learner modeling and scalable personalization [13].

### 2.1. Why TOGAF Matters

TOGAF is well suited to this study because it offers a standards-based method for aligning organizational objectives with IT capabilities across units and systems [14]. In adaptive learning environments, this alignment must also treat uncertainty and information dynamics as explicit architectural concerns. Through shared models, standardized artifacts, and formal governance structures, TOGAF provides a common reference point for technical and non-technical stakeholders who must interpret and act on entropy-informed indicators [15,16].

A key strength of TOGAF is its flexibility: ADM and its artifacts can be tailored to capture uncertainty-aware requirements, entropy-informed KPIs, provenance constraints, and model-risk policies, enabling the architecture to adapt to evolving pedagogical goals, data sources, and infrastructures [14]. TOGAF also promotes reuse of architectural assets, patterns, and building blocks, reducing duplicated effort and controlling the cost of deploying analytics pipelines and streaming infrastructures for real-time learner modeling [17]. Its structured risk-management approach further mitigates data-quality, drift, privacy, and bias risks that are critical for large-scale personalization [9,18].

Accordingly, TOGAF provides the scaffolding to embed entropy-aware analytics into institutional architecture. Through the ADM phases, views and viewpoints, and the Architecture Repository, it identifies where uncertainty arises, preserves and governs information flows, and maintains traceability from stakeholder concerns to implementation decisions. It also integrates with frameworks such as Zachman [19], ITIL [20], and Agile [3]. In this study, these mechanisms make uncertainty measurable and actionable by linking stakeholder uncertainty, metadata, provenance, and entropy-based indicators to architectural artifacts and governance checkpoints [21,22].

### 2.2. TOGAF in Business Enterprises

In organizations, TOGAF has been used to define target architectures, perform gap analyses, and create transformation roadmaps across business, data, application, and technology domains [23]. These uses support stronger business–IT alignment, more robust governance, and improved interoperability. Case studies in utilities, healthcare, and large IT modernization highlight TOGAF’s flexibility in heterogeneous organizational contexts [24,25].

From an uncertainty-oriented perspective, this literature proposes extending TOGAF with uncertainty registers linked to capability maps [26], information flow diagrams enriched with variability and information-gain indicators [27], and governance checkpoints to monitor drift, confidence, and bias in analytics services [28].

### 2.3. TOGAF in Educational Institutions

In higher education, TOGAF has been used for technology-infrastructure planning, application-portfolio rationalization, and data management and governance, all foundational for interoperable, analytics-ready environments [29]. Reported initiatives include standards formalization, consolidation of student, learning, and administrative systems, and governance practices that enhance operational efficiency and data-driven decision-making. These practices also enable the computation and operationalization of entropy-based indicators of learner state, engagement variability, and model confidence in adaptive learning environments [30]. Related case studies show that TOGAF can be tailored to educational settings but also underscore the need to link business architecture more explicitly to data and application components that expose uncertainty and information flow to educators and support services.

Existing TOGAF-based work already offers mature methods for alignment, integration, and governance across enterprise and educational domains [31]. This study builds on that foundation by reframing these methods in terms of information-theoretic observables and treating entropy, uncertainty quantification, and information dynamics as core design and governance concerns within ADM, the Architecture Repository, and the content framework [32,33].

As shown in Table 1, prior TOGAF applications in higher education have focused on planning, interoperability, governance, or reference-model construction, but have not formalized entropy-based observables as reusable architectural artifacts. In contrast, this study extends TOGAF across the full ADM cycle by introducing formally specified uncertainty-aware KPIs, quantifiable uncertainty hot spots, computable data and interface requirements, TRM-aligned analytics infrastructure, and governance mechanisms that use uncertainty reduction and expected information gain for prioritization, migration planning, and implementation control.

Prior TOGAF-in-HEI work has emphasized standards, interoperability, system planning, and technology rationalization but has not treated entropy-based observables as reusable artifacts or systematically linked them to solution packaging, migration sequencing, and governance checkpoints. This study instead contributes: (i) formal entropy-informed KPIs and uncertainty hot-spot metrics; (ii) explicit placement of these indicators across business, information systems, technology, and governance domains; and (iii) a release-planning and ARB workflow that uses uncertainty reduction and expected information gain as decision criteria.

## 3. Methodology

**Figure 1:**
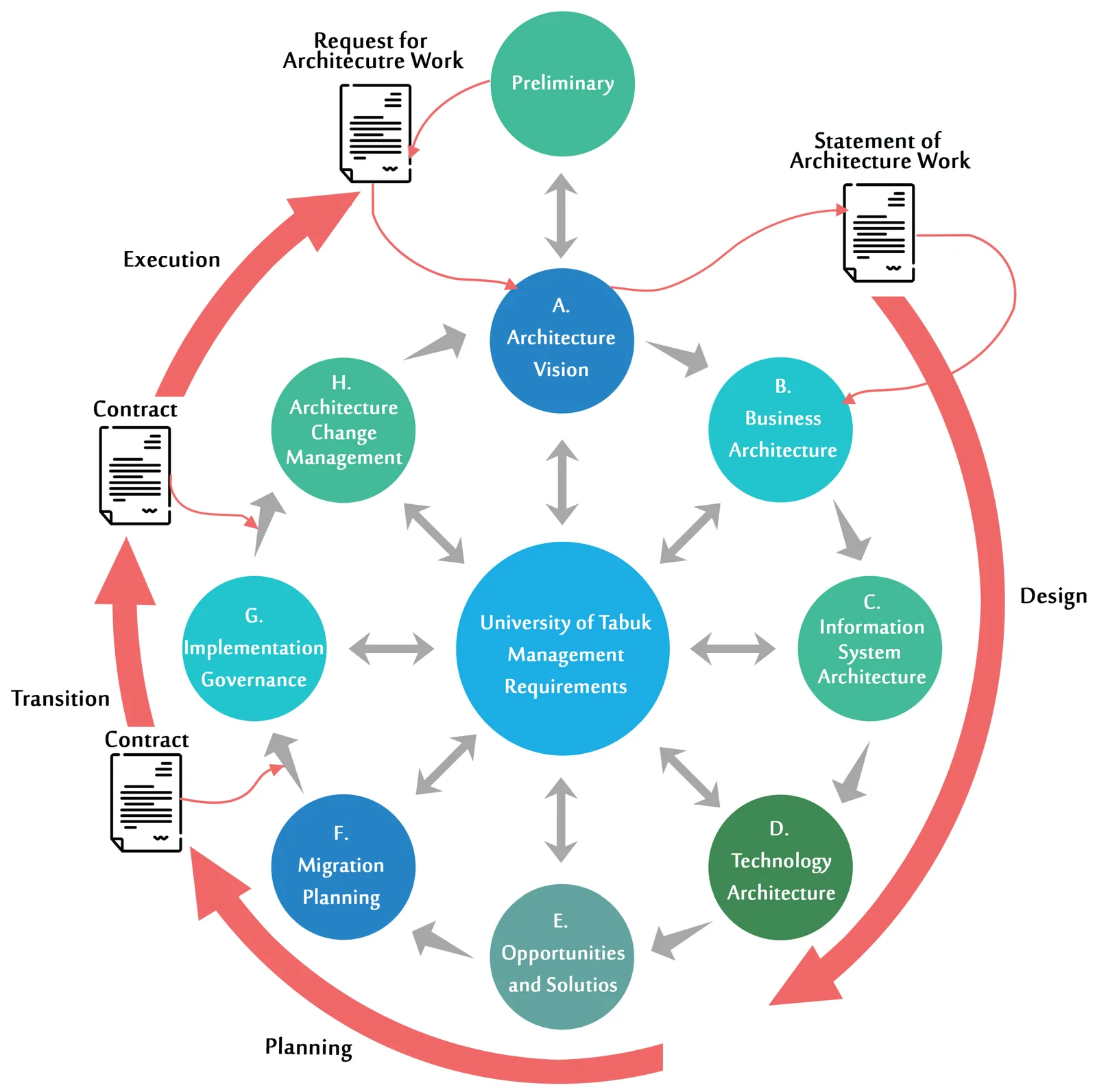
TOGAF Architecture development method (ADM) adapted for adaptive learning, with entropy- and uncertainty-aware checkpoints embedded across phases: (A) architecture vision; (B) business architecture; (C) information systems architecture; (D) technology architecture; (E) opportunities and solutions; (F) migration planning; (G) implementation governance; and (H) architecture change management [10].

We adapt TOGAF’s ADM to UT’s organizational and educational context, embedding entropy- and uncertainty-aware requirements to enable adaptive, personalized learning. The ADM has eight phases: (A) architecture vision, (B) business architecture, (C) information systems architecture, (D) technology architecture, (E) opportunities and solutions, (F) migration planning, (G) implementation governance, and (H) architecture change management, as shown in Figure 1.

Across these phases, deliverables and decision gates are augmented with information-theoretic observables—entropy, information gain, and variability—plus explicit governance criteria. This approach treats epistemic and aleatory uncertainty as primary design concerns, keeping architectural decisions TOGAF-conformant while grounding them in quantitative measures of uncertainty and information dynamics.

The study combines two layers: an architectural layer, where uncertainty-aware requirements are embedded in TOGAF artifacts and governance checkpoints, and an information-theoretic layer, where core observables are formally defined, computed from governed institutional data, and applied to design, prioritization, and planning. This goes beyond architectural description by quantifying uncertainty, tracing its propagation across data and application flows, and incorporating it into migration and governance decisions.

These observables are explicit ARB decision criteria in opportunities and solutions, migration planning, and implementation governance. At each gate, initiatives are evaluated against readiness conditions: traceability to business objectives, availability of canonical data entities, completeness of metadata and provenance, and compliance with security, privacy, and KPI requirements. Release sequencing is driven by expected uncertainty reduction, information gain, and governance readiness, prioritizing initiatives with higher decision value and implementation preparedness.

Entropy-aware artifacts and checkpoints are integrated across the ADM. Phase A defines adaptive learning and data-driven personalization as strategic drivers, sets entropy-informed KPIs, and identifies stakeholder needs for uncertainty visibility in dashboards and decisions. Phase B models capabilities, value streams, and processes to locate uncertainty hot spots and decision points, and maps information flows showing where data are generated, transformed, and consumed.

Phase C covers data and application architectures. The data architecture preserves event-level granularity and shared semantics to compute entropy and information gain across heterogeneous sources. The application architecture integrates learning, advising, and administrative systems to minimize information loss in services supporting near-real-time adaptation, with metadata and lineage enabling traceability and interpretability.

Phase D defines streaming, storage, and analytics platforms for near-real-time exposure of information dynamics. TRM [34] building blocks standardize integration patterns and scalability for uncertainty-aware analytics. Phases E–F prioritize initiatives by expected information gain, anticipated reduction in decision uncertainty, and explicit risk–cost trade-offs, with migration plans favoring incremental releases that expand entropy coverage across critical learner-facing and operational processes.

Phases G–H establish checkpoints for data quality, model-confidence calibration, and drift/bias monitoring. Architectural change is governed through analytics-to-policy feedback loops, maintaining alignment between entropy-aware architectural decisions and UT’s educational objectives.

These extensions are embedded in the TOGAF content framework so entropy-informed KPIs, uncertainty annotations, and information flow mappings become standard, reusable deliverables in the UT Architecture Repository, strengthening cross-phase and cross-project consistency while preserving conformance with the open standard.

### 3.1. Information-Theoretic Formalization Within the TOGAF Adaptation

This TOGAF adaptation makes information theory operational, not just conceptual. Entropy, expected information gain, workflow variability, and uncertainty hot-spot severity are treated as formal observables across the architectural lifecycle and computed from governed UT data pipelines. Phase A defines entropy-aware KPIs as decision criteria tied to stakeholder concerns and institutional objectives. Phase B uses them to locate uncertainty hot spots and decision-critical handoffs where ambiguity, latency, or information loss degrade decisions. Phase C specifies the data models, metadata, lineage, and interfaces needed to preserve event-level evidence and reproducibly compute the indicators. Phase D delivers the secure, scalable streaming, storage, analytics, and observability environment to run and monitor these computations. Phases E–H then apply the metrics to portfolio prioritization, release sequencing, implementation governance, and architectural change management, forming a continuous chain from KPI definition to computable data, operational execution, and governance.

As defined in Section 3.3, the core observables are $H_{\mathrm{pred}}\left(i,t\right)$ , which quantifies uncertainty in learner-state estimates; $\mathrm{EIG}(a|D_{i,t})$ , which measures expected uncertainty reduction from a candidate assessment or interaction; $H_{\mathrm{wf}}$ , which captures dispersion in workflow states, transitions, or paths; and UHS(h) , which combines normalized uncertainty, evidence delay or loss, workflow variability, and decision criticality at a business handoff or decision point. Together, these observables provide a common basis for representing and comparing uncertainty across business, information systems, technology, and governance.

These observables are computed directly from UT’s event-level educational and administrative data, not treated as abstract labels. Predictive entropy comes from posterior learner-state distributions estimated using harmonized evidence (Blackboard activity, assessments, advising interactions, and student records). Expected information gain is the difference between current learner-state entropy and the expected posterior entropy after a candidate assessment or interaction. Workflow variability entropy is computed from the empirical distribution of workflow states, transitions, or paths extracted from service logs. The uncertainty hot-spot score combines normalized uncertainty, latency or information-loss measures, workflow variability, and decision criticality. Because these computations rely on canonical entities, timestamped events, metadata, provenance, and governed interfaces, they are fundamentally architectural rather than purely analytical.

The indicators align with TOGAF domains as follows: Business architecture identifies where uncertainty matters; information systems architecture defines the data and applications to compute it; technology architecture executes the computations securely and at scale; and migration planning, implementation governance, and change management apply the resulting metrics to release, monitor, and enforce the compliance of decisions. Table 2 summarizes this cross-domain positioning in the proposed UT architecture.

### 3.2. Graphics-Enhanced Representation of TOGAF Artifacts

In this study, graphics-enhanced means augmenting standard TOGAF artifacts with lightweight visual annotations that make uncertainty, information flow, and governance relevance explicit. Instead of presenting value chains, content frameworks, and functional decomposition diagrams only as structural views, the figures add uncertainty hot-spot overlays, entropy-informed KPI labels, cross-phase traceability cues, metadata and lineage highlights, and governance markers. This notation keeps familiar TOGAF structures while making uncertainty-aware requirements and decision points easier for architects, advisors, and institutional stakeholders to interpret. Table 3 summarizes the notation used across the figures.

### 3.3. Mathematical Formulation, Assumptions, and Computational Derivation of Entropy-Informed KPIs

To formalize the entropy-informed KPIs in the proposed architecture [35], let $Z_{i,t}$ be learner *i*’s latent state at time *t*, and $p_{i,t}\left(k\right)=P\left(Z_{i,t}=k|D_{i,t}\right)$ the posterior probability that learner *i* is in state *k* given observed evidence $D_{i,t}$ . Here, $D_{i,t}$ may include harmonized event-level data from UT systems such as the LMS, assessment platforms, advising services, and student-information records [36].

For a discrete random variable *X* with probability mass function p(x) , Shannon entropy quantifies uncertainty [37] as follows:

$$H\left(X\right)=-\sum_{x}p\left(x\right)\log p\left(x\right),$$

For two discrete random variables *X* and *Y*, conditional entropy is

$$H\left(X|Y\right)=-\sum_{y}p\left(y\right)\sum_{x}p\left(x|y\right)\log p\left(x|y\right),$$

and mutual information is

I(X;Y)=H(X)−H(X∣Y).

In this study, the expected information gain is used in the same spirit as conditional mutual information since it measures the expected reduction in uncertainty about a latent learner state after a candidate response or interaction outcome is observed.

Based on the posterior learner-state distribution, predictive entropy is defined as

$$H_{\mathrm{pred}}\left(i,t\right)=-\sum_{k=1}^{K}p_{i,t}\left(k\right)\log p_{i,t}\left(k\right),$$

This quantity captures uncertainty in the current learner-state estimate: higher values indicate greater uncertainty, whereas lower values indicate greater confidence. For comparison across models with different numbers of learner states, a normalized form may also be used:

$$H_{\mathrm{norm}}\left(i,t\right)=\frac{H_{\mathrm{pred}}\left(i,t\right)}{\log K}.$$

To evaluate the usefulness of a candidate assessment item or interaction, expected information gain [38] is defined as the expected reduction in entropy [39] after observing a future response. For a candidate action *a*,

$$\mathrm{EIG}\left(a|D_{i,t}\right)=H\left(Z_{i,t}|D_{i,t}\right)-\sum_{r\in R_{a}}P\left(r|a,D_{i,t}\right)\;H\left(Z_{i,t}|D_{i,t},r,a\right),$$

where $R_{a}$ is the set of possible responses or outcomes associated with *a*. Larger EIG values indicate that the candidate action is expected to provide more informative evidence for learner-state estimation and decision support [35].

To make uncertainty hot spots reproducible and comparable across value streams, we define a composite uncertainty hot-spot score for handoff or decision point *h* as:

$$UHS\left(h\right)=w_{1}{\tilde{H}}_{h}+w_{2}{\tilde{L}}_{h}+w_{3}{\tilde{V}}_{h}+w_{4}{\tilde{C}}_{h},$$

where ${\tilde{H}}_{h}$ is normalized predictive entropy, ${\tilde{L}}_{h}$ is normalized information loss or evidence delay, ${\tilde{V}}_{h}$ is normalized workflow variability, and ${\tilde{C}}_{h}$ is the criticality of the affected decision or service outcome. The weights $w_{1},\ldots ,w_{4}$ satisfy $\sum_{i}w_{i}=1$ and reflect governance priorities. Higher UHS(h) values indicate stronger candidates for intervention, integration improvement, or earlier release prioritization.

Workflow variability entropy [40] is used to quantify dispersion in institutional service processes. Let q(j) denote the empirical probability of workflow state, transition, or path *j* in a given business process [41]. Workflow variability entropy is then defined as

$$H_{\mathrm{wf}}=-\sum_{j=1}^{J}q\left(j\right)\log q\left(j\right),$$

Higher values indicate more unpredictable workflows; lower values indicate more stable, repeatable behavior. In the proposed UT architecture, this measure is used mainly in the business architecture and governance layers to identify service workflows with high operational variability and to flag uncertainty hot spots at cross-unit handoffs.

These computations rely on explicit assumptions to ensure repeatability. First, learner and workflow states are treated as discrete variables; continuous measures such as engagement scores, time-on-task, response latency, or risk scores are discretized into governed categories before entropy is computed. Second, calculations use explicit time windows Δt so Blackboard, assessment, advising, and other event streams are aggregated consistently at learner, course, workflow, or institutional levels. Third, workflow variability is computed over fixed observation windows and stable process definitions to preserve comparability across reporting periods. Fourth, each analysis uses a consistent logarithm base so entropy and information-gain values remain comparable across indicators and releases.

These KPIs are computed through a staged pipeline. Event-level data from UT instructional, administrative, and advising systems are ingested and harmonized into canonical learner- and event-level records with timestamps, metadata, and provenance. A learner-state model then estimates the posterior distribution $p_{i,t}\left(k\right)$ over learner states. Predictive entropy is computed from this posterior, and expected information gain is computed as the current entropy minus expected posterior entropy averaged over candidate outcomes weighted by their predicted probabilities. Thus, the architecture defines both the mathematical form of the indicators and their implementation in the institutional data and analytics pipeline.

To compare candidate initiatives in opportunities and solutions and migration planning, uncertainty reduction is the expected decrease in one or more baseline uncertainty measures after an initiative is implemented. For initiative *m* affecting decision points or workflows $S_{m}$ :

$$UR\left(m\right)=\sum_{s\in S_{m}}\omega_{s}\left(U_{\mathrm{before}}\left(s\right)-U_{\mathrm{after}}\left(s\right)\right),$$

where $U_{\mathrm{before}}\left(s\right)$ and $U_{\mathrm{after}}\left(s\right)$ denote the relevant uncertainty measure before and after the initiative, such as predictive entropy, workflow entropy, or hot-spot score, and $\omega_{s}$ denotes the governance weight of decision point or workflow *s*. For comparison across initiatives, a normalized form may also be used:

$$NUR\left(m\right)=\frac{UR(m)}{\sum_{s\in S_{m}}\omega_{s}U_{\mathrm{before}}\left(s\right)}.$$

Initiatives are then compared using normalized uncertainty reduction, expected information gain, governance readiness, and explicit risk–cost considerations, treating uncertainty reduction as a computable quantity rather than a qualitative goal.

### 3.4. Empirical Basis, Data Sources, and Artifact Derivation Protocol

The architectural artifacts in this study are grounded in institutional evidence rather than abstract models. The design draws on: (i) publicly available UT materials, including organizational, strategic, and service information from the university website; (ii) the documented UT system landscape—academic, administrative, research, advising, and reporting platforms such as EREGISTER, MYUT, Blackboard, SRD, ERP, Masar, RDSS, Safeer, CCPS, GMSMS, Daim, and MyServices; (iii) stakeholder requirements and viewpoints from the preliminary and requirements phases; and (iv) input from UT’s data-governance context. The empirical basis is further strengthened by the author’s role in UT’s data-governance unit, which enabled authorized access to additional structural, governance, and system-level information relevant to EA derivation.

The study uses both public and restricted institutional materials. Public sources established institutional structure, service boundaries, and named systems. Additional architecture-relevant materials—such as internal inventories, governance records, integration context, and system-structure details—were reviewed under authorized access and confidentiality obligations and informed the reported artifacts, but are not reproduced due to privacy, security, and confidentiality constraints. Live operational event streams were not used to derive the initial 74 entities and 16 business functions; these are reserved for later pilot and deployment phases.

Artifact derivation followed a staged ADM-aligned procedure. Institutional documents and system descriptions were first organized into an inventory of UT’s primary and supporting activities, then mapped to business capabilities, stakeholder concerns, and value-chain systems. Stakeholder views and requirements were consolidated to refine scope, terminology, and uncertainty-sensitive decision points. FDDs were developed for the identified business functions, and candidate entities were extracted from these diagrams and related system descriptions. Where available, authorized internal materials validated entity boundaries, terminology, and cross-system relationships at an aggregated architectural level. Overlapping entities were then harmonized into canonical data models and registered in the architecture repository with metadata, lineage, and uncertainty-aware instrumentation requirements.

Using this protocol, the 74 entities across 16 business functions reported in Phase C were obtained by translating validated business functions into candidate data objects and harmonizing them into canonical forms with timestamps, provenance, model-confidence fields, and, where needed, event-level granularity. Reproducibility in this study therefore concerns the derivation logic, modeling procedure, and architectural transformation steps, not the unrestricted release of protected institutional materials. Table 4 summarizes the empirical basis and staged derivation used to construct the main architectural artifacts in the proposed UT design.

### 3.5. Research Questions

In accordance with TOGAF guidelines, significant challenges are encountered in the transition from Phase A (architecture vision) to Phase B (business architecture). This transition requires the systematic translation of stakeholder concerns into verifiable and traceable requirements, mapped across the canonical architectural interrogatives: data (what?), function (how?), networking (where?), resources (who?), time (when?), and motivation (why?). In this context, we formulate our research questions so as to explicitly foreground epistemic uncertainty and the dynamics of information flow and evolution.

- (R.Q.1) How can a TOGAF-conformant enterprise architecture design be constructed for UT that embeds entropy- and uncertainty-aware requirements and yields blueprints aligned with institutional needs? Answered in Section 4.1 and Section 4.2.
- (R.Q.2) How can dispersed data from different units be integrated, semantically harmonized, and exposed to compute information-theoretic indicators for near–real-time personalization without access delays? Answered in Section 4.5 and Section 4.6.
- (R.Q.3) How does UT operationalize IT demand planning within opportunities and solutions and migration planning using uncertainty-reduction and expected information gain as prioritization criteria? Answered in Section 4.7.

## 4. Results

We used ADM to design a campus-wide, entropy-aware EA for UT that aligns IT with institutional goals and supports large-scale adaptive learning. This phase turned strategic goals into architectural blueprints and migration roadmaps, explicitly identifying uncertainties and key information flows across academic and administrative domains. Following TOGAF, it organized artifacts, governance checkpoints, and decision milestones across business, information systems, and technology, enabling controlled, future-ready architectural evolution.

### 4.1. Preliminary Phase

**Figure 2:**
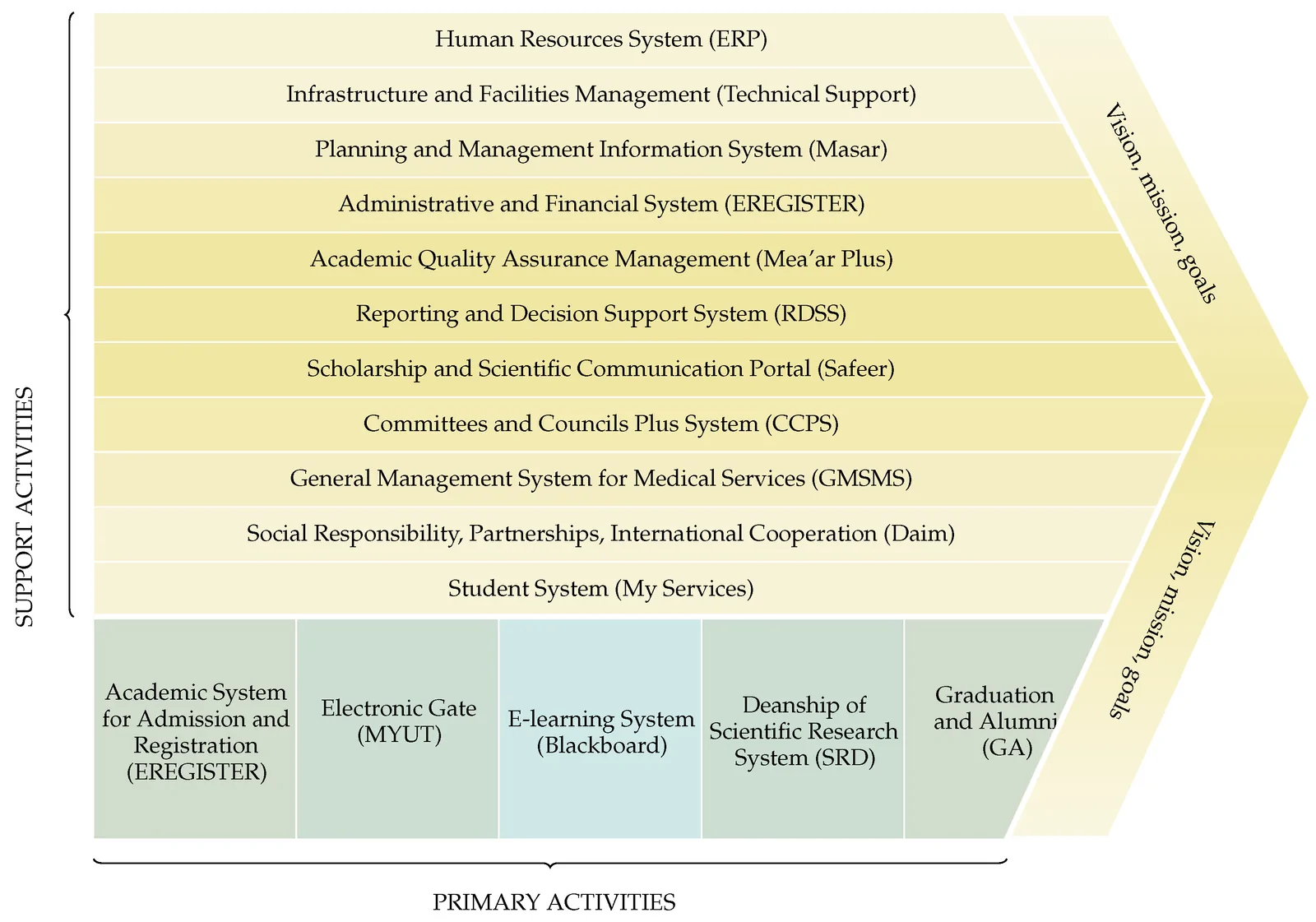
Value chain activities of the University of Tabuk (UT) business process, adapted from Porter’s model, delineating primary (admissions and enrollment, teaching and learning, research, graduation and alumni) and supporting (human resources, infrastructure and facilities, finance and administration, quality assurance, analytics and reporting, partnerships and student services) activities that structure subsequent TOGAF ADM phases [42].

Scope and baseline. We defined UT’s EA scope by mapping primary and support activities with Porter’s value chain [42], establishing the organizational boundary and end-to-end value stream to be supported (Figure 2). This yielded an initial capability and process inventory for later ADM phases and ensured traceability to institutional priorities.

Principles and requirements. Using the TOGAF content framework, we defined architecture principles and high-level requirements to guide design and governance. These treat uncertainty and information dynamics as core concerns by requiring critical services to retain event-level telemetry, expose uncertainty to decision-makers, and maintain auditable provenance for analytical outputs. The resulting artifacts provide a reusable basis for consistent enforcement across initiatives.

Uncertainty and information flow mapping. We mapped uncertainty hot spots at key handoffs—admission-to-enrollment, registration-to-LMS, LMS-to-advising, and research administration-to-reporting. For each, we specified: (i) data-generation points to preserve granularity for entropy and information-gain estimates; (ii) stakeholders needing uncertainty visibility in operational dashboards (e.g., advisors, instructors, registrars); and (iii) minimum metadata and provenance for institution-wide interpretation. These annotations form a baseline for later business, data, and application architecture work to reduce information loss and enable near-real-time personalization.

Stakeholder and capability alignment. This phase aligned stakeholders around a shared, repository-backed view of capabilities and outcomes so later business and information systems architectures stay anchored in UT’s mission and strategic objectives, improving coherence across academic, student-support, and administrative functions for subsequent integration.

The preliminary phase delivered: (i) a value-chain-based EA scope; (ii) entropy-aware architecture principles and requirements embedded in the content framework; (iii) an uncertainty and information flow map showing cross-unit integration points; and (iv) a stakeholder-capability matrix to guide later ADM phases and governance. Together, these form the foundation for business architecture modeling and for defining the data and application services needed to compute and operationalize information-theoretic indicators across UT’s learning ecosystem.

### 4.2. Management Requirements Phases

This phase consolidates stakeholder concerns and business scenarios into traceable architecture requirements for the full ADM cycle. It defines baseline scope, constraints, and success criteria, treating uncertainty and information dynamics as explicit requirements. Each business scenario specifies entropy-aware KPIs, uncertainty hot spots at cross-unit handoffs, and information flow instrumentation to support adaptive learning and near-real-time decision-making. Requirements are stored in the architecture repository using the TOGAF content framework so principles, drivers, and capability needs stay reusable, consistent, and auditable across initiatives.

At UT, a key challenge is consolidating network and asset data while keeping the detail needed for analytics and operational reporting. We address this by combining capability-based planning with TRM building blocks, metadata standards, and lineage policies so event-level data are captured once, governed centrally, and reused across instructional, advising, and administrative systems. This reduces integration risk and enables entropy and information-gain metrics across the learner lifecycle.

The phase delivers: (i) a catalog of business scenarios and stakeholder concerns mapped to views and viewpoints; (ii) architecture principles and non-functional requirements with uncertainty-aware KPIs (e.g., learner-state entropy, assessment information gain, workflow variability); (iii) a register of integration points with required semantics, provenance, and latency budgets; and (iv) governance checkpoints for data quality, model confidence, drift, and bias, enforced during implementation governance and change management.

The activities and systems in Table 5 provide the source of truth for later business, information systems, and technology architecture work. Their integration points and telemetry requirements are defined here to support UT’s analytics and personalization objectives in later ADM phases.

### 4.3. Phase A: Architecture Vision

UT launches its EA program with a university-wide vision that treats uncertainty-aware, information-centric capabilities as core requirements for adaptive learning and personalized education. Following ADM guidance for the vision phase, UT creates EA and data-management functions and a cross-functional team to align architecture, data governance, and analytics with institutional goals and national initiatives such as digital government and Vision 2030. To ensure consistency and reuse across projects, the architecture vision is documented using the TOGAF content framework.

UT’s strategic goals in education, research, community impact, governance, infrastructure, and financial sustainability are translated into architecture drivers, desired outcomes, and measurable decision criteria that guide subsequent ADM phases.

To operationalize the vision, we create core TOGAF artifacts—principles, requirements, capability maps, and baseline/target summaries—and link them to stakeholder views so that concerns, information needs, and uncertainty tolerances remain traceable. These artifacts are stored in the architecture repository to support reuse, governance, and auditability.

Vision deliverables support adaptive learning and information dynamics. First, principles and policies require preserving event-level learning telemetry, exposing model confidence and uncertainty to decision-makers, and enforcing provenance and lineage for interpretability and audit. Second, views reflect the needs of instructors, advisors, program leaders, and administrators, including required uncertainty visibility in dashboards, latency targets for near-real-time interventions, and escalation paths when confidence thresholds are breached. Third, entropy-aware KPIs are standard deliverables: (i) entropy of learner-state distributions; (ii) expected information gain from assessments and interactions; and (iii) variability metrics for service workflows affecting student outcomes. These KPIs become explicit decision criteria for later ADM phases.

The mathematical definitions of these entropy-informed KPIs are provided in Section 3.3. Predictive entropy quantifies uncertainty in learner-state estimation as the Shannon entropy of the posterior learner-state distribution, while expected information gain measures the anticipated entropy reduction after an assessment or interaction, representing the value of that evidence for decision support.

**Figure 3:**
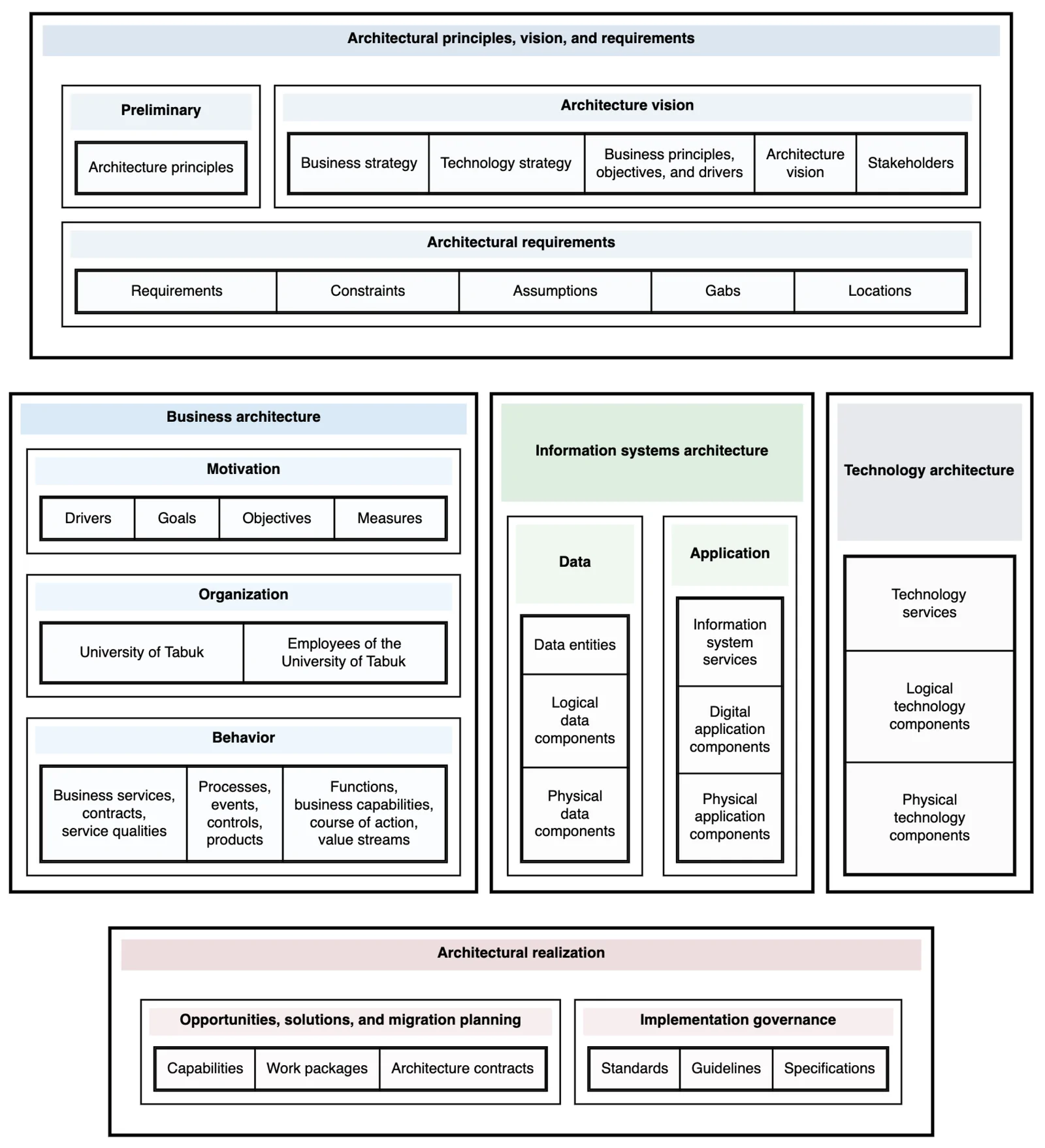
UT’s TOGAF-based architecture content framework, covering principles, vision, requirements, business architecture, information systems architecture, technology architecture, and realization, with entropy- and uncertainty-aware KPIs and governance artifacts embedded across deliverables [43].

This phase also defines the architectural boundary using UT’s value chain and system landscape, along with constraints and success criteria for data quality, security, privacy, uncertainty reduction, and decision effectiveness. These criteria are later verified through implementation governance and change management. Figure 3 summarizes the UT content framework—principles, vision, requirements, business, information systems, and technology architectures, and realization planning. This metamodel clarifies deliverables and relationships among architectural building blocks, improving integration and maintaining consistency as the architecture evolves.

Event-level learner telemetry is collected and used only for defined educational, advising, operational, and governance purposes that directly support UT’s institutional objectives. Personally identifiable student data are limited to authorized operational uses and are not available to analytics, reporting, or decision-support services unless formally justified and governed. Governance dashboards default to aggregate or pseudonymized data, with identifiable learner-level access allowed only for approved roles and intervention scenarios requiring direct student support.

### 4.4. Phase B: Business Architecture

In TOGAF, the business architecture models enterprise motivation, organization, and behavior via services, processes, functions, and qualities. Building on architecture vision, UT’s business architecture aligns capabilities and value streams with uncertainty-aware, information-centric objectives for adaptive learning. As shown in Figure 3, this instantiation maintains traceability from strategic drivers to business services and decision points where uncertainty and information flows must be visible to stakeholders.

The architecture is based on UT’s value chain and system landscape (Figure 2), distinguishing primary academic services from supporting administrative functions. Primary activities include Academic Admission and Registration (EREGISTER), the MYUT portal, Blackboard, SRD, and Graduation and Alumni (GA). Supporting activities include HR (ERP), Infrastructure and Facilities Management, Planning and MIS (Masar), Administrative and Financial Services (EREGISTER), Academic Quality Assurance (Mea’ar Plus), RDSS, Scholarship and Scientific Communication (Safeer), CCPS, GMSMS, Daim, and MyServices.

Each capability and process is annotated with uncertainty-aware business requirements: uncertainty hot spots at cross-unit handoffs (e.g., admission → enrollment, LMS → advising, research → reporting) where latency or information loss degrades decisions; entropy-informed KPIs (e.g., learner-engagement variability, learner-state entropy); and required information flows and provenance so advisors, instructors, and administrators can judge model confidence and act in time. These annotations ensure later data, application, and technology architectures preserve the event granularity and semantics needed for adaptive intervention.

**Figure 4:**
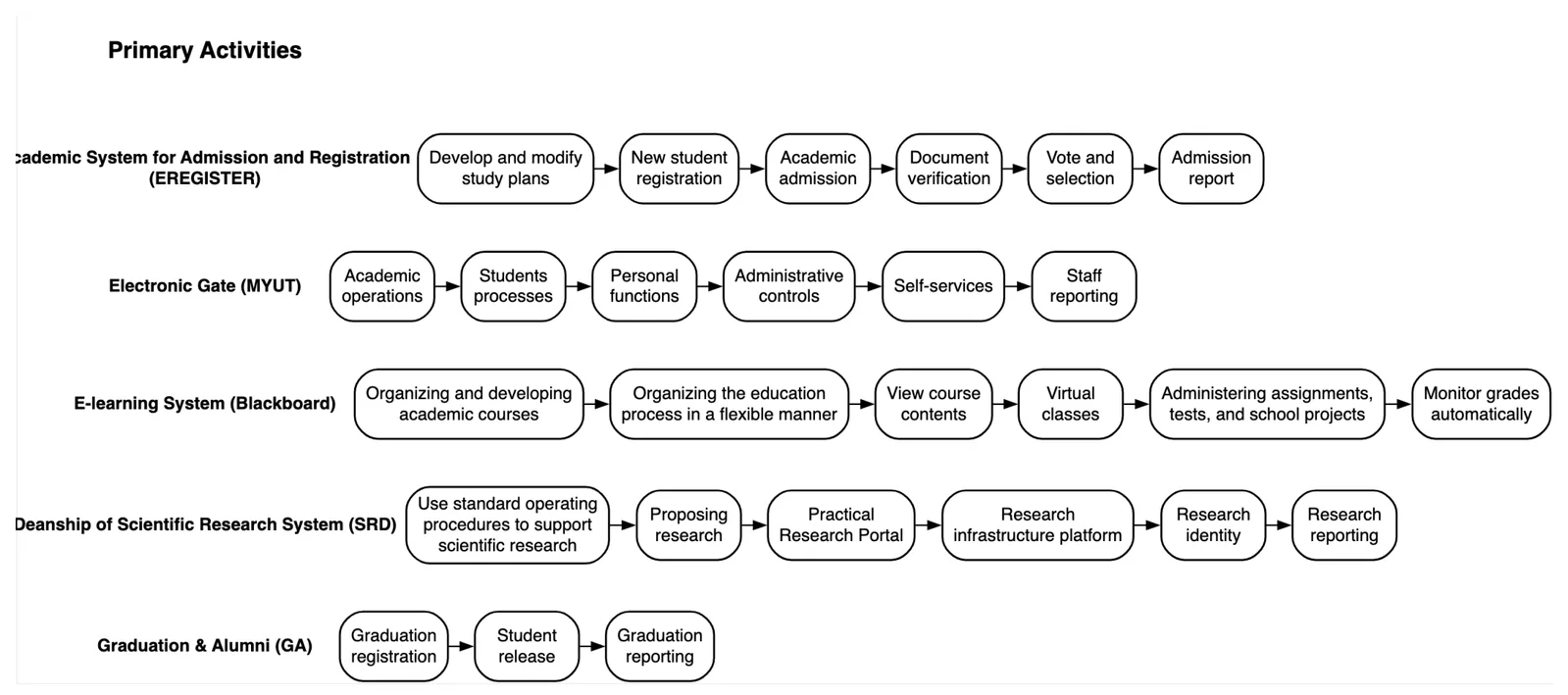
Partial FDDs for UT’s primary activities—admissions and registration (EREGISTER), MYUT, Blackboard, SRD, and graduation and alumni (GA)—showing uncertainty hot spots and entropy-informed KPIs at key handoffs, including admission → enrollment and LMS → advising, to support adaptive-learning use cases and preserve traceability to Phase C data and application requirements.

**Figure 5:**
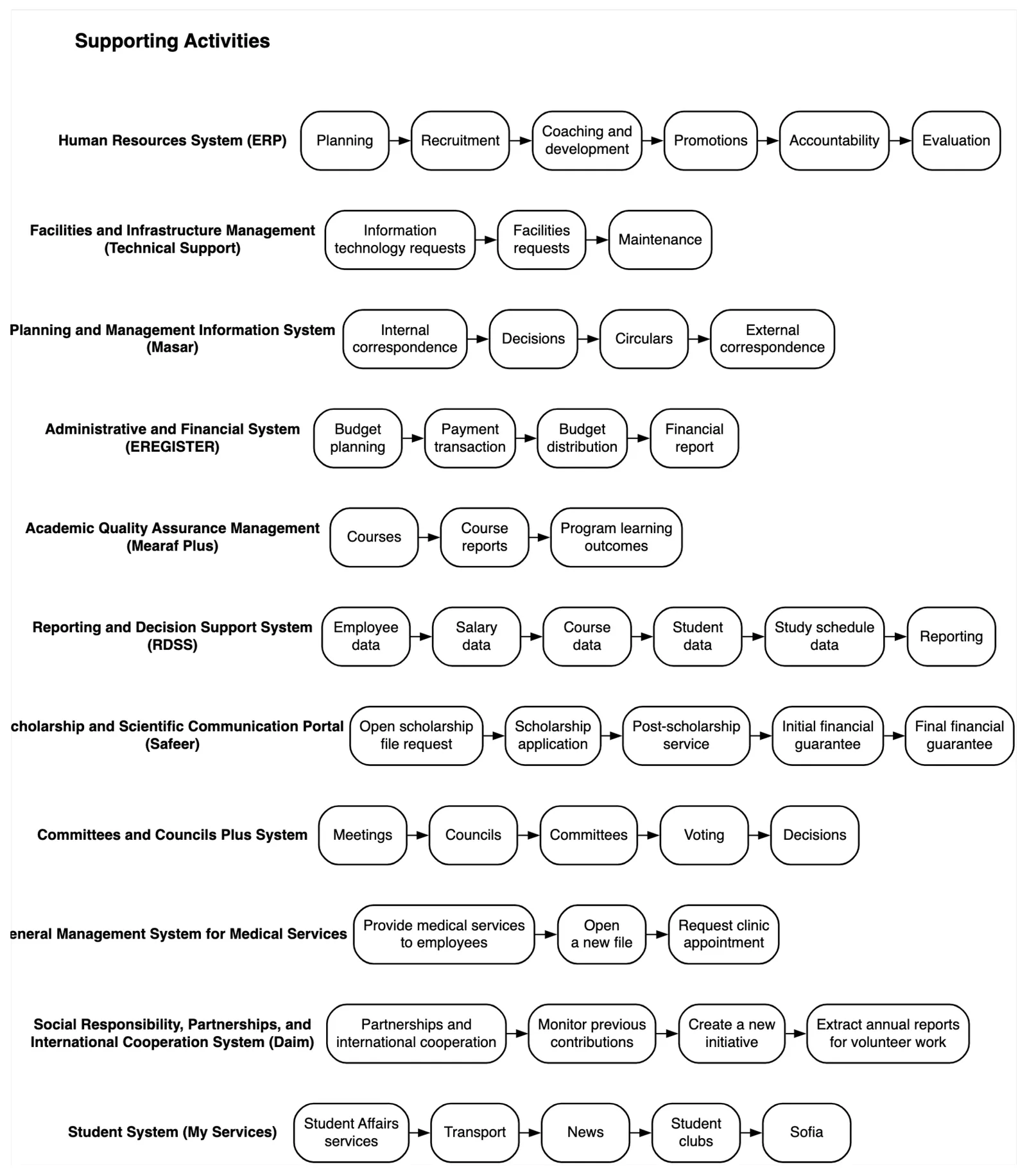
Partial FDDs for UT’s supporting activities, showing the decomposition of value streams into services and subprocesses in Phase B (business architecture), with traceability to Phase C requirements.

To make business behavior explicit, primary and supporting activities are decomposed into services and subprocesses using FDDs. The resulting FDDs (Figure 4 and Figure 5) provide a stable basis for identifying decision points, telemetry requirements, and control policies to be governed in later ADM phases; see also [44].

The business architecture organizes UT’s cross-functional value streams into student enrollment, course delivery and assessment, advising and intervention, research administration, and institutional reporting. Each value stream is defined by business outcomes aligned with UT’s strategic objectives, stakeholder viewpoints specifying uncertainty visibility and latency targets for near-real-time action, and policy requirements for capturing and exposing information-dynamics indicators across units.

Phase B delivers (i) capability maps and value streams annotated with uncertainty hot spots and entropy-informed KPIs; (ii) catalogs of business services, processes, roles, and policies linked to the identified systems; and (iii) viewpoint specifications that formalize stakeholder concerns about uncertainty and information flow. These artifacts guide Phases C and D, keeping integration and analytics designs traceable to UT’s business objectives for trustworthy, personalized education.

### 4.5. Phase C: Information Systems Architecture

**Figure 6:**
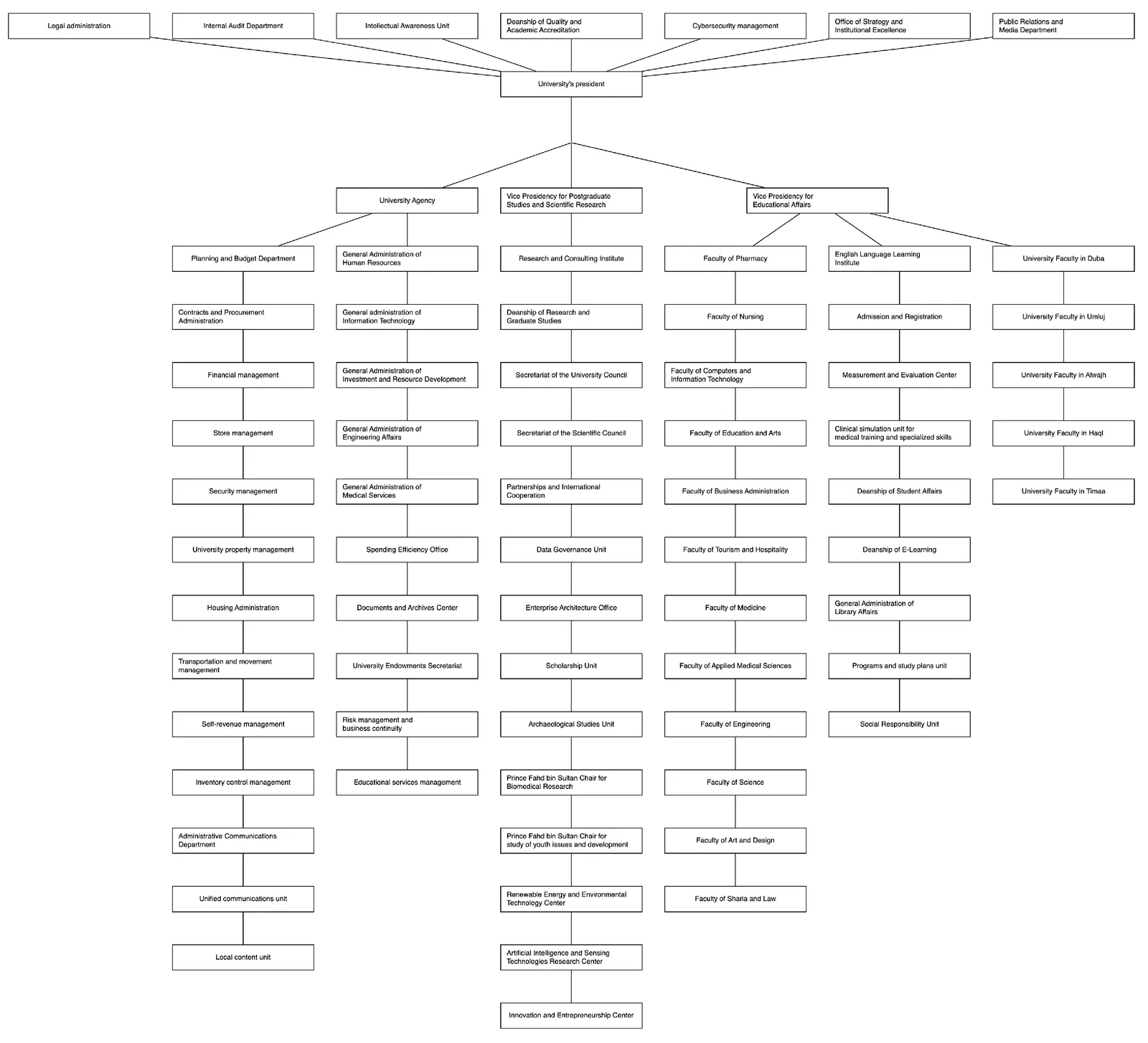
Data–entity diagram for UT spanning primary and supporting activities, with canonical entities and relationships (74 entities from 16 business functions) and uncertainty/entropy instrumentation fields (e.g., event granularity, model confidence, provenance) to support adaptive learning analytics.

Phase C defines UT’s data and application architectures so that information required by business capabilities is captured, governed, and available for adaptive learning and decision support. Using the business architecture and FDDs, we derived entities from existing business functions and organized them into a canonical, entropy-aware data design that preserves event-level granularity for learning analytics and near-real-time intervention. In the UT baseline, 74 entities were identified across 16 business functions (Figure 6), forming the basis for the logical and physical data models and integration with academic and administrative applications.

The data architecture defines canonical models and shared semantics to align datasets across core and supporting activities—admissions and registration, LMS, research, HR, finance, quality, advising, and student services—reducing information loss at cross-unit handoffs. These models, derived from the FDDs, are registered in the architecture repository for reuse. The architecture also specifies metadata, lineage, and data-quality controls for trustworthy analytics, including provenance for assessment events, advising interactions, and administrative transactions, ensuring that uncertainty-aware indicators remain interpretable and auditable.

Uncertainty-aware instrumentation is built into the schema via fields for model confidence, prediction intervals, distribution summaries, assessment- and interaction-level events needed to compute learner-state entropy and information gain over time, and workflow-variability measures. These serve as non-functional constraints on the ingestion and transformation pipelines implemented in Phase D.

Data zones and latency budgets span raw, curated, and feature/serving layers. Raw zones store immutable event logs; curated zones standardize learner, course, program, and service entities; and feature/serving layers power analytics and dashboards for near-real-time adaptation. Latency targets follow Phase B stakeholder needs, such as advisor dashboards refreshing within minutes.

Core applications and services include EREGISTER, MYUT, Blackboard, SRD, ERP/HR, Masar, Mea’ar Plus, RDSS, Safeer, CCPS, GMSMS, Daim, and MyServices. Interface contracts preserve the telemetry and semantics needed for analytics and personalization across these systems.

TRM-aligned integration patterns standardize APIs, messaging, and security to enable scalable, near-real-time data flows from source systems to analytics and decision-support components. Uncertainty-aware services support learner-state estimation, model monitoring, and feedback orchestration by publishing uncertainty and information-gain metrics to advisor and instructor applications and subscribing to evidence streams (e.g., assessment results, activity traces) to refine recommendations. RDSS provides the semantic and visualization layer, exposing entropy-informed KPIs and workflow-variability indicators for governance and academic leadership while preserving lineage to canonical models.

Phase C therefore delivers: (i) canonical data models and dictionaries for 74 entities across 16 business functions; (ii) metadata and lineage specifications, plus data-quality thresholds, that make uncertainty estimates auditable; (iii) application-interface contracts and integration patterns that preserve event granularity and semantics across UT systems; and (iv) an uncertainty-aware analytics and service blueprint showing how entropy, information gain, and variability metrics are operationalized. Table 6 lists the key entropy-informed KPIs, their mathematical definitions, required inputs, computational methods, update cadence, and primary decision uses. Together, these deliverables provide the information backbone for Phase D by enabling streaming, storage, and analytics platforms that meet the latency, reliability, and security requirements for adaptive learning at institutional scale.

Reproducibility of entropy-informed indicators in Phase C depends on preserving computable evidence across UT systems. Canonical entities for learners, courses, assessments, advising interactions, and service events must be consistently defined; events must remain timestamped and traceable; and metadata and lineage must document source, transformation, and use. Interface contracts must also preserve the semantics and granularity needed for downstream analytics. The architecture must expose explicit model outputs—posterior probability distributions, confidence values, and uncertainty annotations—so predictive entropy, expected information gain, and workflow variability can be recomputed and audited from the same governed inputs. Table 6 defines the core entropy-informed KPIs operationally, and Table 2 shows how these indicators align with TOGAF domains in the proposed UT architecture.

#### Application Architecture

The application architecture defines how UT’s applications work together to deliver business capabilities while preserving the granularity, semantics, and provenance needed for adaptive learning and governance. Based on the business architecture and its functional decomposition, domain requirements were translated into application services and interface contracts (Table 7). Applications are modeled as participants in institution-wide information flows that support near-real-time analytics and entropy-informed decision-making, rather than as isolated silos.

Interoperability uses a hybrid integration model that combines canonical, versioned APIs for transactional exchange with event-driven messaging for high-volume telemetry from instructional and administrative processes. This enables learner modeling evidence—assessment events, LMS interaction traces, and advising outcomes—to move across systems with event-level detail preserved. Within this integration fabric, UT’s core systems—EREGISTER, MYUT, Blackboard, SRD, ERP/HR, Masar, Mea’ar Plus, RDSS, Safeer, CCPS, GMSMS, Daim, and MyServices—both supply and consume data from the shared information backbone established in Phase C.

To support uncertainty-aware decision-making, interface specifications are extended to carry uncertainty signals with factual data. Recommendations and alerts include model-confidence scores, prediction intervals, and provenance identifiers; event-level assessment and interaction streams are retained to compute learner-state entropy and information gain over time; and a shared semantic registry ensures indicators remain comparable across academic and administrative units. Latency budgets from Phase B stakeholder viewpoints are bound to these interfaces to enable timely advising and instructional intervention.

The technical framework follows the TOGAF TRM and uses standard building blocks—API gateway, secure messaging and streaming, identity and access management, metadata and lineage services, and observability and monitoring—to reduce integration risk and support scalable evolution. Table 8 lists the infrastructure components needed to streamline UT’s business processes and reliably transport the uncertainty- and entropy-aware payloads defined in this architecture.

The application architecture adds shared services for learner-state estimation, recommendation, and policy orchestration. These services subscribe to instructional and administrative evidence streams, update state estimates and uncertainty, and publish actionable signals to advisor and instructor applications. RDSS serves as the semantic and visualization layer, exposing entropy-informed KPIs and variability metrics to governance and academic leadership while maintaining traceability to canonical data models and Phase C source events.

Within Phase C, entropy-informed KPIs are computed directly from canonical data and application artifacts. Canonical learner, course, assessment, advising, and service-event entities supply state and evidence variables for posterior learner-state estimation. Metadata and lineage specifications ensure that entropy, expected information gain, and workflow variability are reproducible from source events, transformation logic, and timestamped evidence streams. Application-interface contracts preserve assessment outcomes, interaction traces, and confidence annotations so these indicators remain computable and comparable across UT systems.

### 4.6. Phase D: Technology Architecture

The technology architecture implements the information needs defined in the business and information systems architectures by specifying platform services, networks, and security controls that move, store, and process institutional data for analytics and decision support. Following TOGAF’s TRM, it defines core platform principles that standardize data exchange and ensure scalability and governance across heterogeneous systems. Source systems expose client interfaces and data feeds that traverse secure networks into managed data platforms, including data management, metadata management, and business intelligence layers, creating an end-to-end path from transaction to insight for near-real-time decisions. This reference flow underpins the UT platform blueprint and appears in the technology architecture view, which shows source systems, data management, metadata services, and business intelligence tools.

**Figure 7:**
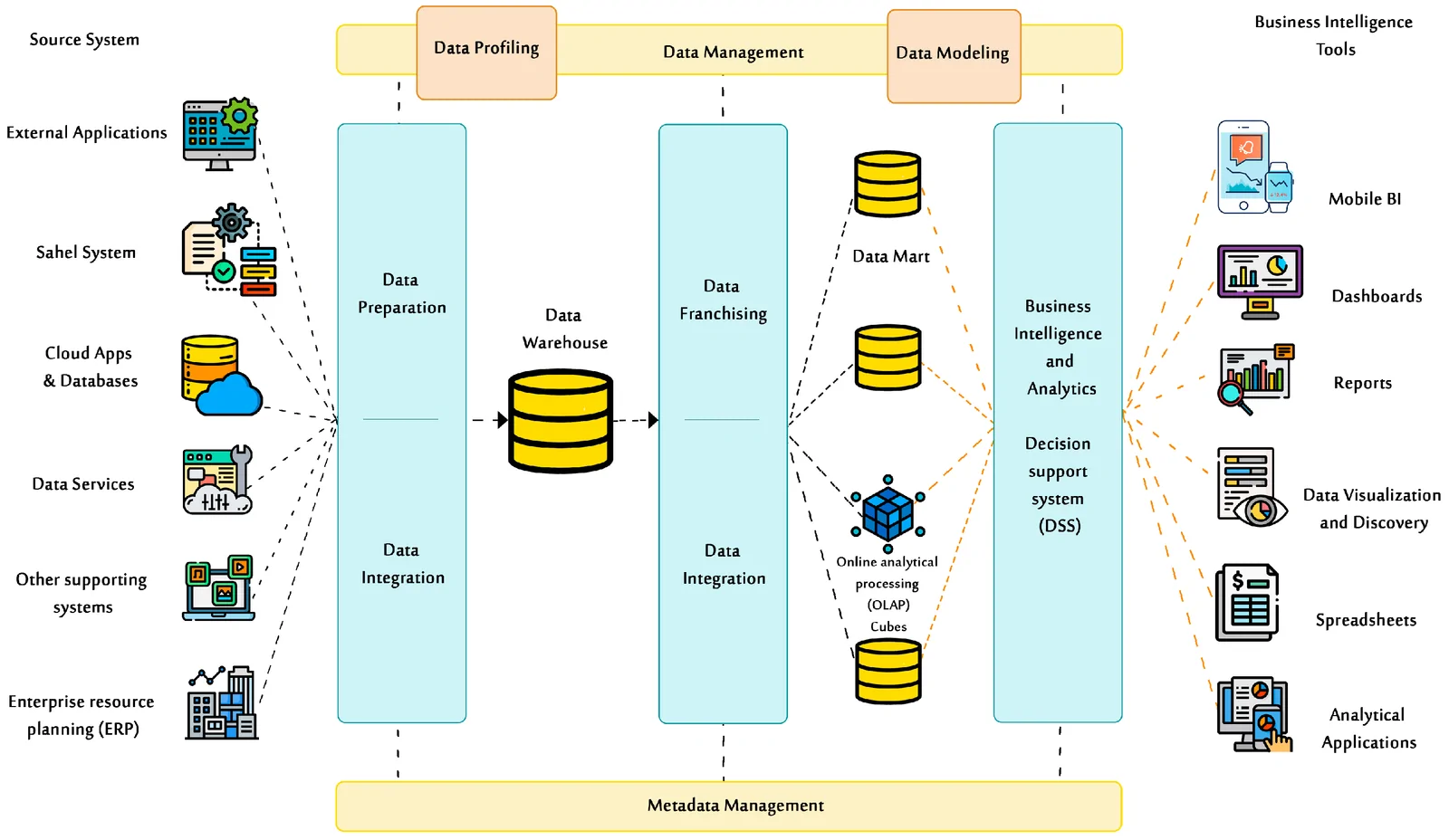
Technology architecture for UT aligned with the TOGAF TRM, showing source systems, data management, metadata services, governance, and analytics components that support uncertainty-aware decision making.

To reduce integration risk and technical debt, TRM building blocks are selected and tailored to UT so that application interfaces from Phase C can be implemented consistently, see Figure 7. This approach enforces common integration patterns and platform services across domains: standardized interfaces for transactional interoperability, streaming and messaging for high-velocity telemetry, and shared security and identity controls. These support current applications and future growth. Using TRM-aligned components enables integration of legacy and new systems while complying with institutional policies for data quality, privacy, and resilience.

The platform design emphasizes trustworthy connectivity and defense-in-depth. Guided by architecture principles from opportunities and solutions, the network is physically and logically segmented to separate transactional paths, with DMZs, VPNs, and perimeter firewalls mediating external access. IDS/IPS, authenticated management access, and encryption preserve confidentiality, integrity, and availability across the data lifecycle. These measures, along with redundant wide-area service providers, maintain the availability and timeliness required for operations and analytics-driven decision support.

At the infrastructure level, UT uses structured gap analysis to compare the current “as-is” platform with the desired “to-be” state. The analysis drives targeted updates to network topology, firewalls, routers, ISP links, switching, storage, IDS/IPS, and VPN services, while retaining suitable server investments and adding management tools to improve observability and operations. This incremental modernization follows TRM guidance and preserves service continuity as capabilities expand.

Phase D puts into operation the information backbone defined in Phase C. Source systems, including the Sahel environment that aggregates many application architecture systems, publish data into governed platforms where metadata, lineage, and semantic harmonization ensure consistent analytics. This stack supports reporting, decision support, and uncertainty-aware analytics for adaptive learning, including entropy-based indicators of learner-state variability and information gain from assessments and interactions. By combining standardized TRM building blocks with secure, scalable network designs, UT ensures accessible, trustworthy data for timely academic and administrative decisions.

Technology architecture does not define entropy-aware KPI semantics; it supplies the infrastructure to compute them reliably and at scale. Streaming pipelines, governed storage, metadata services, and observability components ensure that evidence for entropy and information-gain calculations is ingested with sufficient timeliness, fidelity, and traceability. The technology layer operationalizes—but does not redefine—the indicators specified in the business and information systems architectures.

#### Ethical and Privacy-Preserving Design for Learner Telemetry

Because the proposed UT architecture ingests high-velocity, event-level learner telemetry from systems such as Blackboard, MYUT, EREGISTER, RDSS, and advising services, privacy and ethics are engineered in from the outset—not bolted on as after-the-fact compliance checks. Consistent with the manuscript’s governance model, the architecture operationalizes privacy-by-design and ethics-by-design, hardwiring data protection, accountability, and responsible use into the data, application, and technology layers. This directly reinforces information governance’s mandate to protect confidentiality, integrity, and availability, and delivers on the architecture vision, which elevates security, privacy, provenance, and auditability to first-class, non-functional requirements.

At the ethical level, learner telemetry follows seven principles. First, purpose limitation allows collecting and processing event-level data only for defined educational, advising, operational, and governance purposes aligned with UT objectives. Second, data minimization restricts ingestion and retention to attributes needed for learner-state estimation, service monitoring, and approved decision-support uses. Third, proportionality requires that telemetry granularity, frequency, and use match the educational value and risk of each intervention. Fourth, role-based visibility limits access to uncertainty-aware indicators, learner traces, and alerts to authorized actors who need them, such as advisors, instructors, or designated administrators. Fifth, human oversight remains mandatory for high-stakes decisions, so entropy-informed indicators and risk alerts support rather than replace academic judgment, especially for escalation, intervention, or progression. Sixth, bias and drift monitoring are built into governance so model behavior, confidence, and stability are regularly reviewed. Seventh, auditability and accountability are ensured through repository-backed artifacts, provenance, and traceable decision records across ADM phases.

These principles are implemented with privacy-preserving technologies in the UT target architecture. In analytics zones, learner identifiers are pseudonymized or tokenized so most streaming, feature, and reporting processes use analytics-safe IDs instead of direct personal identifiers. When re-identification is operationally necessary, it is restricted to tightly governed services and authorized workflows. Access to learner-level data and dashboards is controlled by the architecture’s identity and access services (IAM, SSO, MFA, least-privilege roles). This approach aligns with UT’s TRM-based platform services for identity and access management, shared security controls, and secure application integration.

Data in motion and at rest is protected with layered safeguards. Telemetry uses secure, encrypted APIs and messaging, and repositories, feature stores, and analytics components use encryption at rest. PKI-backed certificate and key management enable trusted communication across applications, platforms, and network domains. Network segmentation separates transactional and analytics paths, while DMZs, VPN-based access, perimeter firewalls, and IDS/IPS reduce the attack surface for high-velocity learner data. These controls—including secure messaging, VPN, certificates and keys, firewalling, IDS/IPS, logging, and encryption—are standard elements of the UT technology platform and solution design for uncertainty-aware analytics.

All telemetry for entropy-informed indicators includes metadata, timestamps, and lineage showing source, transformations, and usage context. Events and uncertainty-aware outputs can be centrally signed and logged so that access, processing, and downstream decisions remain auditable. This is crucial in UT’s architecture, where RDSS and dashboard layers expose entropy-informed KPIs to governance and academic leadership; preserved provenance keeps model confidence, information-gain indicators, and workflow variability measures interpretable and reviewable. The architecture also separates identifiable operational data stores from curated analytics zones, and retention controls limit how long high-granularity learner telemetry is kept beyond operational need.

Privacy and ethics are enforced through technical and governance controls. During opportunities and solutions, migration planning, and implementation governance, any initiative using learner telemetry must prove readiness in access control, provenance, privacy compliance, and auditability before release. Ongoing checkpoints monitor data quality, model confidence, drift, and bias so that better adaptivity and reduced uncertainty never compromise student privacy, fairness, or institutional trust. Learner telemetry is thus governed as an institutional asset, and its use for personalization must remain secure, proportionate, explainable, and accountable throughout its lifecycle.

Table 9 summarizes the main ethical and privacy-preserving controls embedded in the UT architecture for handling high-velocity learner telemetry.

### 4.7. Phase E: Opportunities and Solutions

Phase E evaluates implementation options, consolidates architectural changes into solution alternatives, and resolves deficiencies from earlier ADM phases through structured gap analysis. It compares the current information systems and technology landscape with the target architectures from Phases B–D, identifying capability shortfalls and integration bottlenecks that impede UT’s business processes and its goals for adaptive, data-driven education. Aligned with TOGAF, this phase converts gaps into actionable solutions, defines packaging and dependencies, and prepares inputs for migration planning. Table 10 presents the corresponding infrastructure and development solutions for the target state, while Table 11 summarizes application disposition decisions from the gap analysis.

The gap analysis shows that several systems need enhancement or replacement to support required processes. A portfolio review assigns each application a disposition—retain, replace, build new, or retire—aligning investments with institutional priorities and readiness. These dispositions, spanning primary and supporting activities, preserve mature capabilities while requiring updates, integrations, or new solutions where functionality or interoperability fall short of the target state. This structured mapping from “as-is” to “to-be” underpins the modifications in Table 10 and the opportunities in Table 11.

Phase E converts Phase D’s abstract technology-platform principles into an operational design, prioritizing network and security patterns that protect data flows and keep the platform stable during application changes. Key measures include logical and physical segmentation of transaction paths; firewalls, IDS/IPS, DMZ, and VPN configurations; and stronger identity, authentication, logging, auditing, and encryption. Redundant connectivity across service providers supports availability. Together, these measures define a reference integration environment that preserves confidentiality, integrity, and availability and shapes the architectural principles for proposed solutions.

To ensure feasibility and reduce delivery risk, we compare the current and target platforms and define incremental upgrades to network topology, perimeter and routing, switching, storage, IDS/IPS, and VPN services. We retain suitable existing servers and add management tools to improve observability. This plan enables gradual realization of the target architecture without disrupting critical services and underpins the “update/retain/add” decisions in the technology platform analysis.

Solutions are prioritized by value, risk, cost, and their impact on uncertainty and information flow. Each option is assessed for how much it reduces decision uncertainty, improves evidence for learners and operations, and speeds and enriches information for adaptive interventions. Accordingly, solutions that strengthen metadata, enrich canonical data models, and expand near-real-time streams from instructional and advising systems are prioritized earlier, because they enable entropy-informed indicators and confidence-aware decision support across downstream services.

Phase E produces: packaged solutions with dependencies and realization principles; a system-level modification plan aligned with organizational goals; and platform upgrades for scalable, secure integration. These outputs feed migration planning, where they are sequenced into work packages and releases with defined risk and cost profiles, and implementation governance, which monitors adherence to architectural principles—especially data quality, security, provenance, and exposure of uncertainty-aware indicators—throughout delivery.

Building on the gap assessment, UT will modernize its information systems in stages, prioritizing components that most constrain secure, timely, lossless information flow across academic and administrative services. Comparing the current (as-is) and target (to-be) states calls for selective upgrades to network topology, perimeter security, routing, switching, storage, IDS/IPS, and VPN services; retention of fit-for-purpose servers; and new management and observability tools to strengthen operations and reliability. These changes expand hardware, software, and communications capacity and align with TOGAF’s TRM guidance for standardizing integration patterns while maintaining service availability during transition.

To make the platform disposition analysis more decision-relevant, Table 12 reports not only whether a component is updated, retained, or added, but also the architectural criteria and supporting evidence. In the proposed UT architecture, these dispositions are driven by interoperability requirements, governed event-level telemetry, metadata and lineage readiness, low-latency analytics, security hardening, operational continuity, and observability. This makes the table an explicit decision artifact linking baseline–target gap analysis to platform-evolution rationale.

From an educational-analytics perspective, the proposed evolution is more than a capacity increase; it enables near-real-time ingestion, curation, and analysis of high-velocity evidence streams for entropy-informed indicators and uncertainty-aware decision support in adaptive learning. By combining network and security upgrades with governed data- and metadata-management, the target platform reduces time-to-insight while preserving provenance and interpretability, both essential for computing information gain and monitoring variability across learner and service workflows [45].

To develop an information system demonstrably fit for purpose, we synthesize opportunities and solutions from a structured gap assessment, then move to detailed design and development while provisioning the enabling infrastructure summarized in Table 10. This packaging step turns capability shortfalls and integration bottlenecks into implementable changes aligned with the TOGAF TRM, ensuring standardized interfaces, governed data flows, and platform services that support near-real-time, uncertainty-aware analytics across academic and administrative processes. Solution options are thus prioritized by value, risk, and cost, as well as by their expected reduction in decision uncertainty and contribution to information gain across learner and service workflows.

Prior university studies using TOGAF have largely focused on technology architecture, leaving concrete opportunities and solution roadmaps underdeveloped. Earlier efforts adapted TOGAF to universities but did not move beyond technology-layer design or propose realizable solution packages [46,47,48]. In contrast, this work explicitly derives opportunities and solutions from the baseline–target comparison, specifies the platform controls and integrations needed for secure, reliable execution, and connects these to governance criteria for data quality, provenance, and uncertainty-aware indicators. This distinction appears in the modernization plan, which specifies what to retain, update, add, or replace at the application and platform layers to reach the target state.

#### Architecture Review Board Decision Gates for Uncertainty-Aware Prioritization

**Figure 8:**
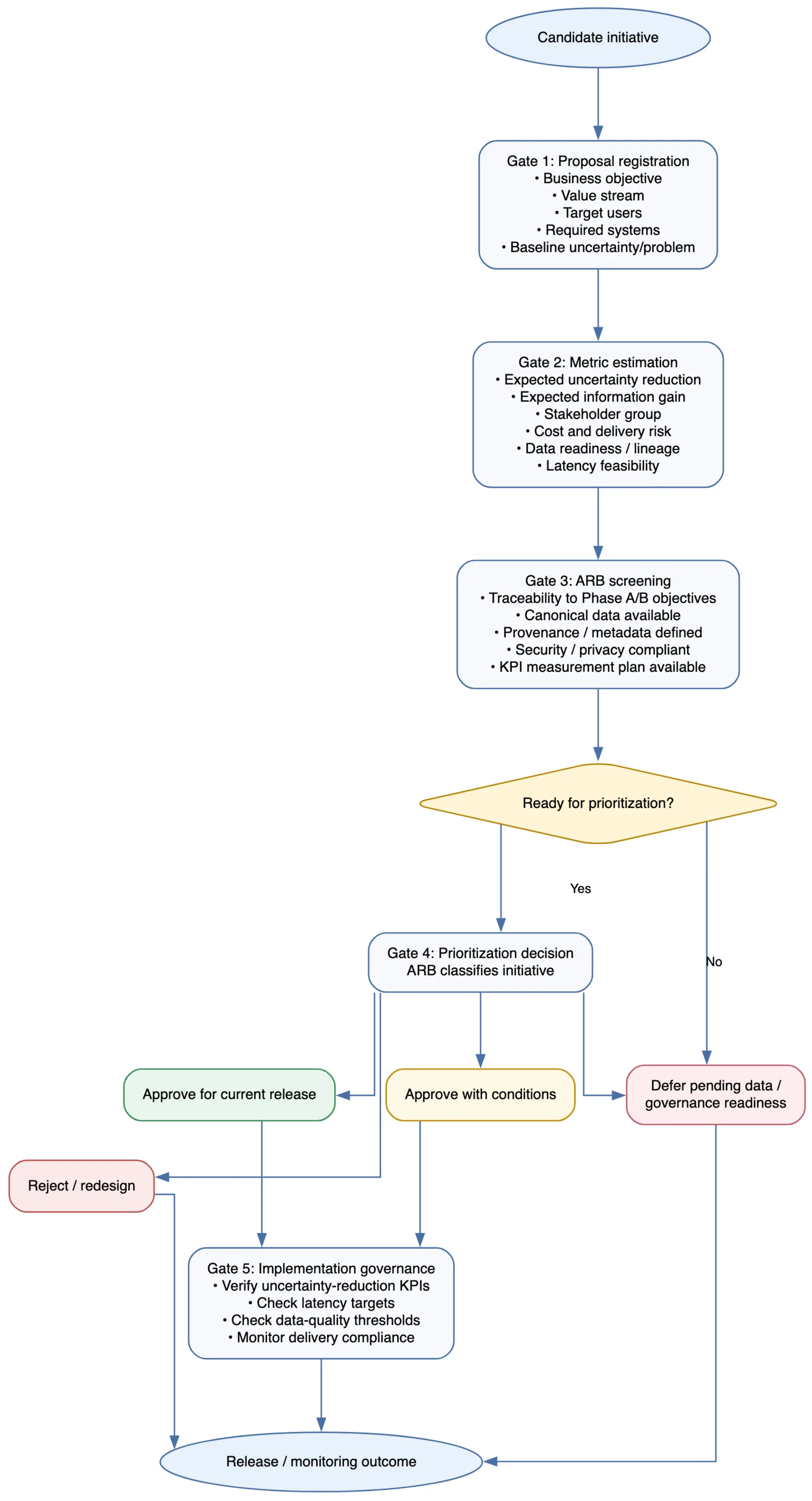
Architecture Review Board (ARB) decision-gate workflow for uncertainty-aware prioritization during opportunities and solutions and migration planning. Candidate initiatives are registered, evaluated using uncertainty- and governance-related criteria, screened against minimum architectural conditions, classified for release planning, and then monitored through implementation-governance checkpoints.

To operationalize uncertainty-aware prioritization, the Architecture Review Board (ARB) uses a staged decision-gate workflow during opportunities and solutions and migration planning, as shown in Figure 8. Each candidate initiative is registered in the architecture repository with its target value stream, stakeholders, affected systems, and baseline decision problem. The proposing team estimates expected uncertainty reduction, information gain, implementation cost, delivery risk, data readiness, and compliance with metadata, lineage, security, and latency requirements. The ARB checks these against minimum readiness criteria and assigns one of four outcomes: approve for the current release, approve with conditions, defer pending data or governance readiness, or return for redesign. Initiatives strongly aligned to institutional objectives, with high expected uncertainty reduction and adequate architectural readiness, are scheduled earlier in the migration roadmap, while those with weak evidence or unresolved governance gaps are deferred until enabling capabilities exist.

An example of uncertainty-aware prioritization is expanding near-real-time Blackboard-to-advising integration for early-warning support at UT. In the baseline architecture, the LMS-to-advising handoff is an uncertainty hot spot: delayed or incomplete evidence limits advisors’ ability to identify and support at-risk students promptly. Under the ARB decision-gate process, this initiative is prioritized because it aligns with adaptive learning objectives, increases timely engagement and assessment evidence, and is expected to reduce uncertainty in learner-state estimation and intervention targeting. As summarized in Table 13, the proposal also shows adequate data readiness, lineage, and security controls, with a moderate implementation effort relative to its anticipated decision value. The ARB would therefore schedule this initiative for an earlier release, with implementation checkpoints to verify latency, data quality, and KPI performance.

Beyond uncertainty reduction and expected information gain, each initiative must meet privacy and ethics readiness requirements—data minimization, role-based access, complete provenance, and compliance with institutional privacy policies—before release approval.

### 4.8. Worked Example: Computing Entropy-Informed Indicators and Using Them in Prioritization

To show how the proposed architecture applies information-theoretic observables, consider a learner whose current state estimate is derived from governed Blackboard activity, prior assessments, and advising evidence in the Phase C canonical data model. Suppose the learner-state model yields the posterior distribution

p=[0.20,0.50,0.30],

over three latent mastery states (low, medium, high). The predictive entropy of this learner-state estimate is

$$H_{\mathrm{pred}}=-\sum_{k=1}^{3}p\left(k\right)\log p\left(k\right)=-\left(0.20\log0.20+0.50\log0.50+0.30\log0.30\right)\approx 1.03,$$

Using the natural logarithm, this value indicates a moderate level of uncertainty in the current learner-state estimate: the posterior is neither maximally uncertain nor sufficiently concentrated to justify a highly confident intervention.

Now consider a candidate Blackboard quiz item or interaction, denoted by *a*, with two possible outcomes: correct $\left(r_{1}\right)$ or incorrect $\left(r_{2}\right)$ . Assume the learner-state model estimates

$$P\left(r_{1}|a,D_{i,t}\right)=0.60,\;P\left(r_{2}|a,D_{i,t}\right)=0.40.$$

If the learner responds correctly, the posterior updates to

$$p\left(\cdot |r_{1},a,D_{i,t}\right)=\left[0.10,\;0.40,\;0.50\right],$$

with entropy

$$H\left(Z_{i,t}|D_{i,t},r_{1},a\right)=-\left(0.10\log0.10+0.40\log0.40+0.50\log0.50\right)\approx 0.94.$$

If the learner answers incorrectly, update the posterior to

$$p\left(\cdot |r_{2},a,D_{i,t}\right)=\left[0.35,\;0.45,\;0.20\right],$$

with entropy

$$H\left(Z_{i,t}|D_{i,t},r_{2},a\right)=-\left(0.35\log0.35+0.45\log0.45+0.20\log0.20\right)\approx 1.05.$$

The expected posterior entropy after administering the item is therefore

$$E\left[H_{\mathrm{after}}\right]=0.60\left(0.94\right)+0.40\left(1.05\right)=0.98,$$

and the expected information gain of the candidate item [39] is

$$\mathrm{EIG}\left(a|D_{i,t}\right)=H_{\mathrm{pred}}-E\left[H_{\mathrm{after}}\right]=1.03-0.98=0.05.$$

This result shows that the item yields a small but positive expected reduction in learner-state uncertainty. In the UT architecture, such values support comparing assessments, prompts, or interactions and prioritizing those expected to provide more informative evidence for advising or instructional adaptation.

## 5. Discussion and Limitations

This discussion synthesizes architecture modeling across the TOGAF ADM to answer R.Q.1–R.Q.3 while aligning enterprise design with uncertainty, information dynamics, and personalization. It links institutional strategy with the TOGAF content framework and repository to create consistent, reusable deliverables, and bases integration and platform decisions on TRM-aligned building blocks to support near-real-time, analytics-driven decision-making across UT’s academic and administrative services.

These deliverables were validated through stakeholder reviews with academic, administrative, and technical participants to ensure they reflected UT’s priorities and operations. Consistency was checked via repository-based traceability to business objectives, data entities, application interfaces, and governance requirements, and by comparing the designs with UT’s current system landscape. Alignment with the baseline–target gap analysis confirmed that the models, solution packages, and migration decisions were coherent, feasible, and appropriately scoped before full pilot deployment.

Although full learner-level personalization was not deployed, the architecture was validated for design and implementation readiness using three mechanisms. First, experts conducted a structured review covering TOGAF conformance, strategic alignment, artifact completeness, uncertainty-aware analytics support, governance readiness, and implementation feasibility. Second, a traceability audit checked consistency from stakeholder requirements and business drivers through capability maps, uncertainty hot spots, canonical data entities, interface specifications, and implementation-governance checkpoints. Third, a limited pilot-readiness exercise using Blackboard, assessment, and RDSS/advising data streams verified that integrated telemetry could compute entropy-informed indicators within defined latency and data-quality constraints.

### 5.1. How Can a TOGAF-Conformant Enterprise Architecture Design Be Constructed for UT That Embeds Entropy- and Uncertainty-Aware Requirements and Yields Blueprints Aligned with Institutional Needs?

In response to R.Q.1, the development of a TOGAF-conformant enterprise architecture that systematically incorporates entropy- and uncertainty-aware requirements begins with a value-chain-grounded scoping exercise and stakeholder alignment in the preliminary and management requirements phases (Section 4.1 and Section 4.2). Porter’s value chain was used to distinguish primary from supporting activities, ensuring that all subsequent architectural artifacts remain explicitly traceable to UT’s institutional mission and strategic objectives.

The architecture vision and business architecture were then specified and governed using the TOGAF content framework, which defines relevant deliverables, formalizes relationships among architectural building blocks, and promotes consistent realization across initiatives. Within this structure, stakeholder views and viewpoints systematically link stakeholder concerns and decision criteria to specific architectural elements. In parallel, a university-wide governance arrangement integrated administrative, academic, and technological perspectives to maintain compliance with reference standards and institutional policies.

Together, these design and review activities demonstrate that the contribution is not merely a conceptual blueprint but a structured enterprise architecture specification whose requirements, deliverables, and governance controls can be systematically inspected and assessed. This is crucial for uncertainty-aware enterprise architecture, where value depends not only on strategic alignment but also on keeping uncertainty-related artifacts explicit, traceable, and governable across the full ADM lifecycle.

### 5.2. How Can Dispersed Data from Different Units Be Integrated, Semantically Harmonized, and Exposed to Compute Information-Theoretic Indicators for Near–Real-Time Personalization Without Access Delays?

In response to R.Q.2, distributed data were semantically harmonized and exposed for computing information-theoretic indicators for near-real-time personalization by combining standardized data models and metadata practices with TRM-aligned technology services (Section 4.5 and Section 4.6). The information systems architecture extracted and structured entities from existing business functions into canonical models, yielding 74 entities across 16 functions to support harmonized integration and analytics. The technology architecture then enabled secure, scalable access by selecting appropriate TRM building blocks and instituting centralized data management and network configurations that preserve availability and timeliness. Together, these decisions reduce redundancy, increase observability, and ensure event-level evidence is captured and shared with governed semantics—prerequisites for computing entropy, information gain, and variability metrics over learner and service workflows for personalization.

These entities were derived from UT’s documented business functions, system/service inventory, and stakeholder-validated functional decomposition diagrams, and then consolidated into canonical models through repository-based harmonization and lineage-aware review.

Within this validation scope, the architecture is evaluated not only by learner outcomes but also by measurable properties such as event-to-dashboard latency, semantic consistency of mapped entities, lineage completeness, event completeness, and KPI reproducibility. These measures align with the proposed information systems and technology architectures, which preserve event granularity, metadata, provenance, and secure streaming paths across Blackboard, advising, RDSS, and related governed platforms.

### 5.3. How Does UT Operationalize IT Demand Planning Within Opportunities and Solutions and Migration Planning Using Uncertainty-Reduction and Expected Information Gain as Prioritization Criteria?

For R.Q.3, UT’s IT demand planning is embedded in the opportunities and solutions phase and carried into migration planning (Section 4.7). Following TOGAF, it uses gap analysis, continuous assessment of current and target architectures, and prioritization of initiatives by alignment with institutional objectives, explicitly considering risk and cost. In our tailored approach, prioritization also reflects expected reductions in decision uncertainty and contributions to information gain across key value streams, emphasizing uncertainty management.

At the portfolio level, disposition analysis (retain, replace, develop new, decommission) and comparative evaluation of technology platforms (update, retain, augment) translate architectural gaps into executable work packages and platform evolution steps that are sequenced to avoid disrupting critical services. This structured packaging and phasing—supported by network segmentation, DMZ/VPN perimeters, IDS/IPS deployment, identity security hardening, and provider redundancy—stabilizes the execution environment during integrations and anchors demand planning in demonstrable architectural improvements.

Prioritization is implemented through an explicit ARB decision-gate workflow that links uncertainty reduction, expected information gain, and governance readiness to release decisions. The workflow shows how candidate initiatives are checked against minimum readiness conditions before being approved, conditionally approved, deferred, or returned for redesign. A concrete UT example—near-real-time Blackboard-to-RDSS/advising integration for early-warning support—illustrates how high expected decision value can justify earlier release despite implementation complexity.

This governance view is reinforced by the Methodology section, which formalizes uncertainty reduction as a computable quantity and defines expected information gain mathematically, tied to canonical event streams and governed interfaces. Prioritization is thus grounded in a repeatable information-theoretic layer rather than only architectural narrative.

The discussion has three implications. First, centering governance on the content framework and repository improves consistency of architectural outputs, accelerates reuse, and reduces time-to-value for analytics and decision support. Second, TRM-consistent platform services provide a common fabric for transactional APIs, streaming telemetry, metadata/lineage, and security, shortening the path from transaction to insight and enabling near-real-time information for advisors, instructors, and administrators. Third, explicitly linking business drivers to data, application, and technology designs strengthens institutional adaptability: as uncertainty-aware indicators mature, their semantics, lineage, and performance constraints remain traceable to business outcomes and earlier ADM governance checkpoints. These implications align with documented benefits of TOGAF-aligned practice and our baseline–target comparisons.

This work is an enabler, not an end state. The blueprints, models, and platform choices provide the infrastructure for entropy- and uncertainty-aware analytics at scale, but lasting value depends on implementation governance and change management that monitor data quality, model confidence, drift, and bias, and align evolving educational priorities with architectural constraints. Future work will add operational dashboards and feedback loops so that uncertainty reduction and information gain become core decision criteria across academic and administrative processes.

To complement the architectural results and reflect the study’s non-deployment scope, we also evaluated the proposed design from an architecture-validation perspective.

### 5.4. Architecture Validation and Practical Readiness

Although this study does not report learner-level pilot outcomes, the architecture was validated at the enterprise-design level through artifact completeness, institutional traceability, and implementation readiness. Based on UT’s actual system landscape and value chain, it produced canonical data and application artifacts for 74 entities in 16 business functions, defined baseline-to-target gap resolutions, and specified governance checkpoints for release sequencing and implementation control. The study therefore offers design-stage empirical evidence of feasibility and institutional fit, while learner-level operational validation is intentionally deferred to future pilots.

Table 14 defines the study’s validation scope, separating current evidence from future empirical evaluation. It shows that the manuscript delivers design-stage validation—covering institutional scope, data and integration readiness, technical feasibility, and governance preparedness—via TOGAF artifacts, gap analysis, and implementation planning. Learner-level operational effects are deferred to a later phase, to be evaluated through staged pilots and outcome-based assessments once the architecture is deployed in representative UT services.

At the architecture level, validation used three complementary mechanisms. First, enterprise architecture and TOGAF specialists, UT academic stakeholders, and UT IT and data-governance stakeholders reviewed the proposed design. Using structured criteria—strategic alignment, TOGAF conformance, artifact completeness, support for uncertainty-aware analytics, governance readiness, and implementation feasibility—they assessed the architecture’s formal coherence and its institutional relevance and adoptability within UT.

Second, a traceability-focused validation was applied across the architecture lifecycle. Requirements and business drivers were checked for consistency with business capabilities and uncertainty hot spots, then traced to canonical entities, metadata and lineage specs, application-interface contracts, and implementation-governance checkpoints. Because the adaptation is anchored in the TOGAF content framework and Architecture Repository, this audit confirms that uncertainty-aware KPIs and information flow requirements stay linked to their original institutional objectives rather than becoming isolated analytics artifacts.

Third, the architecture’s practical readiness was assessed through a limited pilot and feasibility proof centered on a representative UT integration scenario: near-real-time Blackboard event streams and assessment outcomes feeding advising and RDSS dashboards for early-warning support. This scenario is appropriate because the LMS-to-advising handoff is a hotspot for uncertainty in the business architecture; this is also because Blackboard, RDSS, metadata and lineage services, and low-latency analytics are core to the information systems and technology architectures. Within this scope, the manuscript does not claim validated learner outcomes; instead, it defines measurable architectural properties to assess whether the entropy-aware design improves operational readiness and analytic reliability.

Table 15 summarizes architectural metrics—event-to-dashboard latency, semantic consistency of mapped entities, lineage completeness, event completeness, and KPI reproducibility. Together, they show whether a limited pilot can demonstrate that integrated Blackboard, assessment, and advising streams sufficiently support governed computation and delivery of predictive entropy, expected information gain, and related uncertainty-aware indicators.

Together, these practices produced a campus-wide architectural blueprint and implementation roadmap whose structure, governance, and artifacts follow TOGAF, tailored to UT’s specific needs and constraints. The manuscript also notes that full learner-level operational validation is planned for a later research phase, using staged pilots and outcome-focused evaluation after the architecture is deployed in representative UT services.

### 5.5. Future Technical Roadmap and Research Design

Although the entropy-informed KPIs are formally specified and computable in the proposed architecture, their learner-level validation in operational UT systems is future work. This study did not deploy or validate learner-level entropy measures or mutual-information-based feature selection in live university systems, nor did it assess how uncertainty reduction affects outcomes such as achievement, retention, or time-to-mastery. In addition, the effectiveness of the specified privacy-preserving controls in real student-data workflows has not yet been empirically tested.

Although full learner-level outcome validation is future work, the manuscript now provides architecture-level validation via expert review, traceability auditing, and a limited pilot-readiness assessment of integrated Blackboard–assessment–advising data streams. This narrows the validation gap by showing that the design has already been examined as a structured, reviewable, and measurable architectural artifact, even though outcome-level educational evaluation is not yet complete.

Future work will follow a staged roadmap aligned with the UT architecture. Phase 1 will establish operational readiness by integrating high-priority event streams from Blackboard, EREGISTER, MYUT, and advising services into the governed analytics environment, while validating metadata completeness, lineage, pseudonymization, access control, and latency. Phase 2 will pilot Blackboard-to-RDSS/advising integration for early-warning support, computing predictive entropy, expected information gain, and workflow variability from live events and exposing them via decision-support dashboards. Phase 3 will evaluate the pilot through a prospective design (e.g., staged rollout, quasi-experimental comparison, or A/B testing) to assess architectural and operational effects. Evaluation will use system-level metrics (event completeness, dashboard latency, KPI reproducibility, model-confidence stability) and service-level indicators (advisor uptake, intervention turnaround time, and selected student-support outcomes). This roadmap progresses from architectural readiness to operational validation while complying with UT’s governance, privacy, and risk-management constraints.

Table 16 presents the technical roadmap from architecture readiness to operational validation in the UT environment. The three phases are as follows: (1) securing data and governance across core systems; (2) piloting entropy-aware analytics via Blackboard-to-RDSS/advising integration; and (3) evaluating workflows in a prospective study using architectural and service-level metrics. This staged plan defines the deployment sequence, target systems, validation focus, and example outcome measures for follow-up studies.

Future work will follow a staged validation plan aligned with the current architecture. Stage one will assess the operational readiness of core UT systems—Blackboard, RDSS, advising workflows, and related governed data services—by checking data completeness, lineage, access control, and dashboard latency. Stage two will pilot entropy-aware analytics in high-priority contexts, starting with Blackboard–RDSS/advising integration for early-warning support. Stage three will evaluate these workflows via prospective designs (e.g., staged rollout, A/B testing, bandit-based evaluation) and report architectural and service outcomes, including uncertainty-aware KPI stability, advisor uptake, intervention timeliness, and key student-support indicators. All stages will comply with UT’s risk, privacy, and cost-governance requirements so that adaptivity and personalization do not compromise robustness, operational stability, or institutional trust.

The manuscript now clearly distinguishes what has been validated at the current design stage from what must still be demonstrated in future operational deployment. This strengthens the contribution’s credibility by showing that the architecture already has expert-reviewed, traceable, and measurable design-stage support, while maintaining appropriate caution about later learner-level and outcome-level claims.

## 6. Conclusions

This study defined a TOGAF-based enterprise architecture for the University of Tabuk (UT) and, across ADM phases, showed how a standards-based blueprint can: (1) design an architecture tailored to UT’s needs; (2) integrate heterogeneous, formerly siloed data through standard models, metadata management, and interoperable components; and (3) support IT demand planning via continuous gap analysis, risk assessment, and cost-benefit evaluation. The results are primarily architectural and governance-focused: a value-chain-based scope, functional decomposition artifacts, canonical data and application models, TRM-aligned technology design, uncertainty-aware KPIs, prioritization criteria, and implementation-governance checkpoints. Together, these deliver an integrated, institution-wide information system architecture that is responsive, extensible, and aligned with UT’s academic, research, and administrative goals.

Beyond these demonstrated artifacts, the architecture also establishes institutional and technical foundations for future entropy-informed analytics. By consolidating data governance and creating a shared architecture repository, it enables the capture and orchestration of rich, time-resolved learner and system interaction data, supporting future modules for quantifying uncertainty, modeling information flow, and enabling adaptive interventions at scale. At this stage, however, these are enabled future capabilities rather than validated learner-level outcomes.

Embedding the ADM’s opportunities and solutions and migration planning activities into governance cycles enables UT to iteratively reduce decision-making uncertainty. The mechanisms used for gap analyses and portfolio prioritization can then be extended to manage information dynamics as new instructional services and analytics pipelines are introduced. The study’s contribution is a formally specified, uncertainty-aware enterprise architecture framework with associated decision and governance artifacts, while learner modeling, content sequencing, feedback optimization, and outcome-level adaptive-learning effects are reserved for later implementation and evaluation phases.

Several limitations remain. Although the entropy-informed KPIs are now formally specified and computable within the proposed architecture, their learner-level empirical validation in operational UT systems is still pending. The manuscript now includes architecture-level validation, traceability auditing, and pilot-readiness assessment, but full learner-level outcome validation is incomplete. Future work will use staged pilots and prospective evaluations in representative UT services to convert architectural readiness into operational evidence of adaptive-learning impact.

## Figures and Tables

**Table 1:** Comparative positioning of prior TOGAF-in-HEI studies and the present work.

Study	Context	Highest TOGAF Coverage	B/D/A/T/Gov Coverage	Roadmap/ Migration	Uncer-Tainty/Entropy Formalization	Main Delta Relative to This Study
Higher-education EA planning study [6]	University planning	ADM-based planning	Partial B/D/A/T; limited Gov	Limited	No	Planning-focused; no entropy-aware KPIs, hot spots, or governance metrics.
College enterprise IS planning study [29]	College EIS	Planning/design	Partial B/D/A/T; limited Gov	Limited	No	Covers enterprise planning, but not formal uncertainty-aware artifacts or roadmap logic.
Smart campus blueprint study [30]	Smart campus	Blueprint/ architecture design	Mainly A/T; limited Gov	Limited	No	Provides a campus blueprint, but not formal information-theoretic indicators or governance use.
Education-sector data governance study [31]	Education governance	Governance/data governance	Strong Gov/D; limited full ADM coverage	No	No	Strong governance basis, but no entropy formalization or migration prioritization.
HEI information-systems reference model studies [32,33]	HEI reference modeling	Reference model	Primarily D/A reference structure	No	No	Reference-model focus; does not operationalize entropy-aware metrics across ADM phases.
Present study	University of Tabuk	End-to-end ADM adaptation (A–H)	Integrated B/D/A/T/Gov	Yes	Yes	Adds formal entropy-aware KPIs, uncertainty hot spots, computable data/lineage artifacts, uncertainty-driven prioritization, and governance checkpoints.

**Table 2:** Positioning of information-theoretic indicators across TOGAF domains.

TOGAF Domain	Role of Indicators	Main Artifact	Example Use at UT
Business Architecture	Identify uncertainty hot spots and decision-critical workflows	Capability maps, value streams, viewpoints	Flags LMS → advising and admission → enrollment handoffs where delayed or incomplete evidence weakens decisions.
Information Systems Architecture	Define computable data and application representations	Canonical data models, metadata, interface contracts	Preserves event-level Blackboard, advising, and student-record data so entropy and information gain can be computed consistently in RDSS and analytics services.
Technology Architecture	Enable secure, timely, and scalable computation	TRM-aligned platform, streaming, storage, security controls	Uses API and event streaming, metadata and lineage, IAM, encryption, VPN, and IDS/IPS to transport and protect telemetry for near-real-time analytics.
Opportunities and Solutions/Migration Planning	Prioritize initiatives by uncertainty reduction and information gain	Gap analysis, work packages, release roadmap	Ranks initiatives such as Blackboard → RDSS integration earlier when they improve evidence availability for early warning and advising.
Implementation Governance/Change Management	Monitor KPI thresholds, drift, bias, and compliance	Governance checkpoints, review gates, audit records	Checks data quality, latency, model confidence, and privacy/security readiness during delivery and architectural evolution.

**Table 3:** Visual notation used in the graphics-enhanced TOGAF artifacts.

Graphic Element	Meaning	Use in the UT Architecture
Annotated handoff or node	Uncertainty hot spot	Marks business or system handoffs where ambiguity, latency, or information loss degrades decision quality, such as LMS→advising and admission→enrollment.
KPI label/callout	Entropy-informed indicator	Shows where predictive entropy, expected information gain, workflow variability, or related metrics are attached to a process, value stream, or architectural component.
Cross-phase traceability cue	ADM linkage	Indicates how a business concern, hot spot, or KPI is carried from vision and business architecture into information systems, technology, and governance artifacts.
Metadata/lineage emphasis	Auditability and interpretability	Highlights where provenance, timestamps, semantic mappings, and governed data definitions are required for reproducible KPI computation.
Governance marker/checkpoint	Review or control point	Indicates where ARB gates or implementation-governance checks assess readiness, compliance, confidence, drift, or release suitability.
Layered architectural grouping	TOGAF domain structure	Preserves the separation among business, information systems, technology, and realization/governance domains while keeping uncertainty-aware overlays interpretable.

**Table 4:** Empirical basis and artifact derivation protocol.

Input Source	Examples at UT	Use in Artifact Derivation	Output Artifact
Public institutional documents	Strategic plan, organizational structure, service descriptions, public system-facing pages	Defined institutional scope, strategic drivers, and primary/supporting activities	Value-chain scope, architecture principles, baseline business context
System and service inventory	EREGISTER, MYUT, Blackboard, SRD, ERP, Masar, RDSS, Safeer, CCPS, GMSMS, Daim, MyServices	Mapped business functions to existing systems and integration points	System landscape, application mappings, integration candidates
Stakeholder requirements and viewpoints	Academic, administrative, technical, and advising concerns consolidated in early ADM phases	Refined business scenarios, decision points, uncertainty-visibility needs, and latency constraints	Requirements catalog, viewpoints, uncertainty hot spots, KPI needs
Authorized internal governance and system-structure materials	Restricted inventories, governance records, integration context, internal structural details	Validated terminology, entity boundaries, and cross-system relationships under confidentiality obligations	Refined architectural mappings, validation of canonical entity structure, governance alignment
Functional decomposition and business modeling	Primary/supporting activity decomposition, FDDs, business services, roles, and processes	Extracted candidate entities, relationships, and business-to-data traceability	Business Architecture artifacts, candidate entities, traceability links
Repository harmonization and governance review	Canonical modeling, metadata review, lineage specification, terminology reconciliation	Merged overlapping entities and added provenance, timestamps, and uncertainty-aware fields	74 canonical entities, metadata/lineage specifications, interface contracts

**Table 5:** UT business activities and their corresponding information system solutions.

Activity	Business Solution	Information System Solution
Academic Admission (EREGISTER)	Study plans, student registration, admission, document verification	EREGISTER System
Electronic Gate (MYUT)	Academic data, guidance, digital library, scheduling, personal data	MYUT System
E-learning (Blackboard)	Course organization, virtual classes, assignments, grade monitoring	Blackboard System
Scientific Research (SRD)	SOPs, application needs identification	Research Portal, Infrastructure, Identity
Facilities Management (Technical Support)	Infrastructure design, asset management	Technical Support System: IT, Facility, Housing Requests
HR Management (ERP)	HR systems, hiring, training, performance	ERP System: Promotions, Communication, Skills, Administration
Transaction Management (Masar)	Correspondence, decisions, planning, transactions	Masar System
Financial System (EREGISTER)	Requirements analysis, system integration	EREGISTER System
Quality Assurance (Mea’ar Plus)	Program planning, document management, assessment	Mea’ar Plus System
Decision Support (RDSS)	Data management for employees, salaries, courses, students	RDSS System
Scholarship Portal	Scholarship, communication, sabbatical requests	Linked with Safeer System
Committees System (CCPS)	Committees management, topics discussion, voting	CCPS System
Medical Services (GMSMS)	Medical services for employees, appointments	GMSMS System
Social Responsibility (Daim)	Monitor contributions, initiatives, partnerships, reports	Daim Platform
Student Services	Services including transport, buffets, news, clubs	MyServices

**Table 6:** Operational definition of entropy-informed KPIs.

Item	Predictive Entropy	Expected Information Gain	Workflow Variability Entropy
Definition	$-\sum_{k}p\left(k\right)\log p\left(k\right)$	$H_{\mathrm{before}}-E\left[H_{\mathrm{after}}\right]$	$-\sum_{j}q\left(j\right)\log q\left(j\right)$
Inputs	Learner posterior probabilities	Current posterior + candidate response model	Workflow event logs and transition counts
Derivation	Directly from posterior state distribution	Expected entropy reduction over outcomes	From empirical workflow-state/path distribution
Cadence	Per learner update	Per assessment or intervention step	Daily or weekly
Use	Confidence display, early warning	Assessment selection, intervention priority	Process monitoring, hotspot detection

**Table 7:** Application modules supporting UT’s primary and supporting activities, including EREGISTER, MYUT, Blackboard, SRD, and GA for primary services, and ERP, Technical Support, Masar, Administrative and Financial systems, Mea’ar Plus, RDSS, Safeer, CCPS, GMSMS, Daim, and MyServices for supporting services.

No	Business Function	Application	Application Code
PRIMARY ACTIVITY			
1	Academic system for admission and registration (EREGISTER)	-Develop and modify study plans -New student registration -Academic admission -Document verification -Vote and selection -Show report	EREGISTER.1 EREGISTER.2 EREGISTER.3 EREGISTER.4 EREGISTER.5 EREGISTER.6
2	Electronic gate (MYUT)	-Academic data and study schedules -Academic guidance -Digital library system -Scheduling integrated lecture halls -Personal and administrative data -Self services -Export reports	MYUT.1 MYUT.2 MYUT.3 MYUT.4 MYUT.5 MYUT.6 MYUT.7
3	E-learning system (Blackboard)	-View course contents -Virtual classes -Administering assignments, tests, projects -Monitor grades automatically	Blackboard.1 Blackboard.2 Blackboard.3 Blackboard.4
4	Deanship of scientific research (SRD)	-Practical Research Portal (new) -Research infrastructure platform -Research identity	SRD.1 SRD.2 SRD.3
SUPPORTING ACTIVITY			
5	Infrastructure and facilities management (Technical Support)	-Information technology requests -Facilities requests -Faculty housing system	TS.1 TS.2 TS.3
6	Human resource management (ERP)	-Academic promotions system -Communication system -My Skills -Direct faculty members -My card -Holding an employee accountable -Professional certificates	ERP.1 ERP.2 ERP.3 ERP.4 ERP.5 ERP.6 ERP.7
7	Administrative and planning transactions management system (Masar)	-Internal correspondence -Decisions -Circulars -External correspondence -Development planning -Transaction system	Masar.1 Masar.2 Masar.3 Masar.4 Masar.5 Masar.6
8	Administrative and financial system (EREGISTER)	-Budget management -Employee Administration Finance -Administration Management Education -Cost of Management Faculty -Salary management -Financial reporting	EREGISTER.7 EREGISTER.8 EREGISTER.9 EREGISTER.10 EREGISTER.11 EREGISTER.12
9	Academic quality assurance management (Mea’ar Plus)	-Planning of programs -Standard for managing documents -Internal monitoring and assessment	Mea’ar Plus.1 Mea’ar Plus.2 Mea’ar Plus.3
10	Reporting and decision support management system (RDSS)	-Employee data -Salary data -Course data -Student data -Study schedule data	RDSS.1 RDSS.2 RDSS.3 RDSS.4 RDSS.5
11	Scholarship and scientific communication portal (Safeer)	-Internal and external scholarship requests -Requests for scientific communication -Sabbatical applications	Safeer.1 Safeer.2 Safeer.3
12	Committees and councils plus system (CCPS)	-Managing committees and councils -Presenting and discussing topics -Voting on committees and councils issues	CCPS.1 CCPS.2 CCPS.3
13	General management system for medical services (GMSMS)	-Providing medical services to university employees -Open a new file -Request an appointment at one of the clinics	GMSMS.1 GMSMS.2 GMSMS.3
14	social responsibility, partnerships and international cooperation (Daim)	-Partnerships and international cooperation -Monitoring previous contributions -Create a new initiative -Extract annual reports for volunteer work	Daim.1 Daim.2 Daim.3 Daim.4
15	Students services application (MyServices)	-Transport -Buffets -News -Student clubs system	MyServices.1 MyServices.2 MyServices.3 MyServices.4

**Table 8:** Technical Reference Model (TRM) within TOGAF.

No	TOGAF TRM Component	Component Description
1	Infrastructure Applications and Shared Services	Enterprise-wide services: identity and access, API gateway, messaging/event bus, content/document services, scheduling, observability, and catalog/metadata/lineage. Includes the data and analytics platform (repositories, stream/processing engines, model registry, feature/serving) to enable entropy/uncertainty analytics with auditable provenance.
2	Business Applications	Domain systems (e.g., admissions/registration, LMS, research admin, HR, finance, QA, advising, student services, reporting/DSS) that produce/consume data for personalization and governance via canonical APIs and event streams.
3	Application Platform and Operating System Services	Runtime stack—operating systems, containers, orchestration, and middleware—providing scalability, reliability, and security; enforces non-functional requirements (latency, availability, integrity) for near–real-time analytics and decision support.
4	Network and Security Services	Core network and security: naming/time, transport/web protocols, secure mail/file services, VPN, certificates/keys (PKI), firewalling, IDS/IPS, and load balancing; delivers trusted, policy-compliant connectivity across applications, platforms, and data planes.
5	Communications Infrastructure	Physical/logical connectivity: campus LAN/WLAN, data-center and cloud interconnects, WAN/Internet links, optical backbones; supplies bandwidth, resilience, and QoS for telemetry, interactive workloads, and streaming pipelines across sites.

**Table 9:** Ethical and privacy-preserving controls for learner telemetry in the UT architecture.

Concern	Architectural Control	Technology/ Mechanism	UT Application
Identity exposure	Pseudonymized analytics identifiers	Tokenization/ pseudonymization	Learner records from Blackboard, MYUT, and EREGISTER are pseudonymized in analytics zones and governance dashboards.
Unauthorized access	Role-based access control	IAM, SSO, MFA, least-privilege roles	Advisor, instructor, and administrator access to RDSS and related dashboards is restricted by role and authentication level.
Data interception	Protected transport channels	TLS, VPN, PKI-backed certificates	Telemetry, APIs, and event streams between UT systems and the analytics platform are encrypted in transit.
Infrastructure attack	Defense-in-depth network security	Segmentation, DMZ, firewalls, IDS/IPS	Transactional and analytics paths are isolated across the UT platform and protected at the perimeter and internal network layers.
Accountability	Traceable processing and access	Central logging, signed events, metadata, lineage	Access to learner telemetry and downstream use in RDSS or analytics services is auditable from source event to dashboard output.
Over-retention	Controlled retention and separation of stores	Retention/deletion rules, zone separation	Identifiable operational data are separated from curated and analytics-ready stores in the lakehouse and governed by metadata policies.

**Table 10:** Current and target application and infrastructure solutions for UT, aligned with TOGAF principles and uncertainty-aware personalization objectives.

No	Infrastructure	Plan	Development	Architectural Principle
1	Security, Hardware, Network Topology	Segment critical/analytics paths; isolate model/feature stores; least-privilege access; DMZ/VPN for external exposure; encrypt data/streams to protect entropy-aware telemetry	Routers/firewalls/IDS–IPS for segmentation; IAM/MFA and centralized logging; TLS-at-rest/in-transit; signed events and provenance to ensure integrity of uncertainty estimates	Data Security and Integrity
2	Data Network	Provider redundancy; QoS for low-jitter telemetry; time synchronization for information-dynamics tracking; near–real-time access to analytics streams	Multi-homing/SD-WAN; SLA-backed latency/jitter; NTP/PTP clock discipline; traffic shaping for streaming pipelines computing entropy/information gain	Service Availability
3	Software Development	Modularize services; externalize commodity apps; internalize governance for analytics; embed uncertainty calibration and A/B/bandit testing	API-/event-first integration; CI/CD and MLOps for model/feature registries; rollout gates using expected information gain; observability for feedback loops	Modularity and Evolvability

**Table 11:** Disposition of UT information systems based on gap assessment and alignment with the target architecture; a checkmark (✓) denotes the selected disposition.

	Status	Retain	Replace	Develop	New	Remove
System						
EREGISTER			✓			
MYUT			✓			
Blackboard		✓				
SRD					✓	
Technical Support			✓			
ERP		✓				
Masar				✓		
Mea’ar Plus		✓				
RDSS			✓			
CCPS					✓	
GMSMS			✓			
Daim			✓			
MyServices				✓		
Graduation and Alumni (GA)				✓		
Safeer		✓				
Library Systems (ILS/Discovery)		✓				
Research Data Repository (RDR)					✓	
Learning Analytics Platform (LAP)					✓	
API Gateway/Integration Platform (iPaaS)					✓	
Identity and Access Management (IAM/SSO)			✓			
Data Lakehouse/MDM					✓	
Unified Mobile App (Student/Staff)					✓	
Chatbot/Virtual Assistant					✓	

**Table 12:** Technology platform disposition analysis with justification criteria and supporting evidence.

Platform Component	Disposition	Justification Criteria	Supporting Evidence in UT Context
Network topology	Update	Segmentation, telemetry isolation, resilient routing	Needed to separate transactional and analytics paths and support secure, low-latency evidence flows.
Firewall	Update	Stronger perimeter control, analytics protection, policy compliance	Required to protect governed data flows and externalized services supporting uncertainty-aware dashboards.
Router	Update	Capacity, routing reliability, integration scalability	Needed to improve connectivity and support increased cross-system traffic from APIs and event streams.
Server	Retain	Existing capacity remains fit for purpose	Current server investments remain usable while higher-priority upgrades focus on networking, storage, and observability.
ISP connectivity	Update	Availability, redundancy, latency stability	Provider redundancy and improved connectivity are needed for reliable near-real-time analytics and service continuity.
Switching	Update	Throughput, telemetry transport, internal reliability	Required to sustain higher-volume institutional traffic and governed streaming pipelines.
Storage	Update	Scalable ingestion, analytics retention, provenance support	Needed for event-level data retention, metadata/lineage support, and analytics-ready storage layers.
IDS/IPS	Update	Threat detection, security hardening, governance assurance	Required to protect sensitive telemetry and strengthen defense-in-depth across integrated services.
VPN	Update	Secure remote/system access, protected data transport	Needed for trusted external and administrative access to integrated platforms and governed services.
Management tools	Add	Observability, monitoring, operational control	Added to improve system visibility, latency monitoring, and support architecture-level validation metrics.
Management access	Add	Secure administration, auditability, controlled operations	Added to strengthen authenticated administrative access and traceable management actions.

**Table 13:** Example ARB decision-gate criteria for uncertainty-aware prioritization.

Criterion	ARB Question	Example Evidence (Blackboard → RDSS)	Decision Implication
Strategic alignment	Supports UT priorities?	Improves early warning and advising; aligned with adaptive learning goals.	Prioritize earlier release.
Uncertainty reduction	Will it reduce decision uncertainty?	Timely LMS activity and assessment data reduce ambiguity in identifying at-risk students.	Higher priority.
Expected information gain	Does it add useful evidence?	Adds quiz, submission, login, and participation signals for learner-state updates.	Favors early approval.
Data readiness/lineage	Are data and lineage ready?	Canonical IDs, timestamps, and lineage rules are available for RDSS ingestion.	Approve if complete; otherwise defer.
Security/privacy	Are controls adequate?	Role-based access, logging, and governed student-data handling are defined.	Proceed if compliant; otherwise conditional/defer.
Cost/delivery risk	Is effort acceptable?	Moderate integration effort; limited disruption to existing systems.	Supports phased approval.
Release recommendation	What is the outcome?	High fit and value; moderate risk; adequate readiness.	Approve for current release.

**Table 14:** Validation evidence provided in this study and deferred empirical validation.

Validation Dimension	Evidence Provided in This Study	Section/Artifact	Deferred Future Validation
Scope coverage	UT value chain, system inventory, business functions mapped	Phases A–B	Expansion to additional units/services
Data/integration readiness	74 entities, canonical models, metadata, lineage, interface contracts	Phase C	Live pipeline testing and KPI reproducibility
Technical feasibility	TRM-aligned platform, security, streaming, observability design	Phase D	Operational performance and latency testing
Governance readiness	ARB gates, release prioritization, implementation checkpoints	Phases E–G	Release execution and compliance monitoring
Learner-level impact	Not evaluated in current study	Limitation	Pilot studies, A/B or bandit evaluations, outcome analysis

**Table 15:** Proposed measurable validation criteria for entropy-aware architectural deployment.

Property	Definition	UT Measurement Example
Latency	Elapsed time from source event to dashboard or analytics availability	Time from a Blackboard event to RDSS dashboard refresh.
Semantic consistency	Degree of conformance to canonical entities and shared semantics	Percentage of records mapped correctly to canonical learner, course, and assessment entities.
Lineage completeness	Traceability of KPI outputs back to source events and transformations	Percentage of entropy/EIG outputs linked to source systems, timestamps, and transformation steps.
Event completeness	Presence of required fields in captured telemetry	Percentage of Blackboard and advising records containing timestamps, actor identifiers, and action types.
KPI reproducibility	Stability of recalculated KPIs from the same governed inputs	Consistency of recomputed entropy and expected information gain values using identical source data and model outputs.

**Table 16:** Future technical roadmap and research design for operational validation.

Item	Phase 1	Phase 2	Phase 3
Technical objective	Establish operational data and governance readiness	Deploy pilot entropy-aware analytics for early warning	Evaluate operational and decision effects
UT systems involved	Blackboard, EREGISTER, MYUT, RDSS, IAM/SSO, Data Lakehouse/MDM	Blackboard, RDSS, advising workflows, Learning Analytics Platform	Blackboard, RDSS, advising services, selected courses/programs
Research design/validation focus	Validate ingestion, pseudonymization, lineage, access control, and latency readiness	Pilot Blackboard-to-RDSS/advising integration and test KPI computation in live workflows	Staged rollout, quasi-experimental comparison, or A/B evaluation of entropy-aware support
Example metrics	Event completeness, lineage coverage, access compliance, dashboard latency	Predictive entropy stability, expected information gain, workflow variability, advisor usage	Intervention turnaround time, advisor response rate, alert precision, selected student-support outcomes

## Data Availability

Public institutional materials used in this study are available through the University of Tabuk’s website. Additional internal structural, governance, and system-level materials were accessed under authorized institutional roles and used only for enterprise architecture derivation and validation. These restricted materials are not publicly shared because they are subject to privacy, security, and confidentiality obligations. Derived architectural artifacts and methodological descriptions are reported in the manuscript.

## Abbreviations

The following abbreviations are used in this manuscript:

A/B	A/B testing
ADM	Architecture Development Method
API	Application Programming Interface
CCPS	Committees and Councils Plus System
CI/CD	Continuous Integration/Continuous Delivery
DSS	Decision Support System
DMZ	Demilitarized Zone
DoDAF	Department of Defense Architecture Framework
EA	Enterprise Architecture
EIA	Enterprise Information Architecture
ERP	Enterprise Resource Planning
GA	Graduation and Alumni
GMSMS	General Management System for Medical Services
HEIs	Higher Education Institutions
HR	Human Resources
IAF	Integrated Architecture Framework
IAM	Identity and Access Management
IDS/IPS	Intrusion Detection System/Intrusion Prevention System
ILS	Integrated Library System
iPaaS	Integration Platform as a Service
IT	Information Technology
ITIL	Information Technology Infrastructure Library
ITSM	IT Service Management
KPIs	Key Performance Indicators
LAN	Local Area Network
LAP	Learning Analytics Platform
LMS	Learning Management System
MDM	Master Data Management
MFA	Multi-Factor Authentication
MIS	Management Information System
MLOps	Machine Learning Operations
NTP	Network Time Protocol
PKI	Public Key Infrastructure
PTP	Precision Time Protocol
QA	Quality Assurance
QoS	Quality of Service
RDR	Research Data Repository
RDSS	Reporting and Decision Support System
SD-WAN	Software-Defined Wide Area Network
SLA	Service-Level Agreement
SOPs	Standard Operating Procedures
SRD	Deanship of Scientific Research System
SSO	Single Sign-On
TAFIM	Technical Architecture Framework for Information Management
TLS	Transport Layer Security
TOGAF	The Open Group Architecture Framework
TRM	Technical Reference Model
UT	University of Tabuk
VPN	Virtual Private Network
WAN	Wide Area Network
WLAN	Wireless Local Area Network
